# Supplementary material for: Growth rules for the repair of Asynchronous Irregular neuronal networks after peripheral lesions
Source: PLoS Comput Biol. 2021 Jun 1;17(6):e1008996. doi: 10.1371/journal.pcbi.1008996 (PMC8195387; doi:10.1371/journal.pcbi.1008996)
Supplement: Attachment — Submitted filename: review.pdf [file pcbi.1008996.s001.pdf]

Review: Growth Rules for the Repair of Asynchronous Irregular Neuronal Networks after Peripheral Lesions

General remarks

The present study proposes a simple yet qualitatively significant change to the MSP model, proposed by Butz et al, that provides a generic framework to describe post-lesion repair. They apply this framework to peripheral lesioning of a balanced AI network aimed at modeling cortical activity.
In addition to the change in the MSP model, they also include I->E STDP plasticity and strength-dependent deletion of synapses to their simulation, then assess numerically how both plasticity mechanisms must work together to generate a partial recovery.
The rationale behind the evolution of post- and pre-synaptic structures for both collective and single-cell dynamics also seems clear and consistent, even though limited experimental and numerical evidence is provided as to why other possibilities should not be considered.
Required code to reproduce the result is provided using free open-source licenses.

I believe that this work represents an interesting contribution and a useful, logical advancement for studies on structural plasticity.
I would support its publication in PLOS CB provided one major question is answered and some restructuring is performed on the manuscript, including the addition of precision/discussions (notably a more in-depth comparison with previous studies).

The one major issue that I have with this study concerns the stability of the network activity with respect to the rules chosen for structural plasticity (SP).
Indeed, the authors only looked at the influence of structural plasticity for 500 s in the control state.
This seems too short compared to the typical timescales over which the changes are occurring during the recovery (several thousand seconds).
Furthermore, most growth curves are intrinsically unstable on their own, making the reason for the overall stability hard to understand, especially as it is not discussed at all (though, to be fair, the non-trivial case of inhibitory neurons was also ignored by Butz et al. in the

original model [33]).

Besides this major issue, I feel that there is a need for more thorough justification of several modeling choices and their consequences:

A. Are the choices related to the growth curves really ensuring the stability of the activity?
B. Was the final increase in synchrony not expected given the chosen parameters?
C. Why was STDP limited to I->E and instead of (for instance) scaling of excitatory synapses?
D. Structural changes are very fast compared to the biological values mentioned in the MSP paper [33] (72 days over the whole timecourse) and the experimental studies that are cited [6, 9] (several days). The necessity of speeding up simulations is understandable (it is also argued in [33]) and it is probably sufficient for the timescales of neuronal activity/STDP and SP to occur on "sufficiently different" timescales to achieve "reasonable modeling". However that "sufficient difference" should certainly be discussed if not proven, especially given the fast growth of inhibitory axons in the current study.

More specific remarks and questions regarding precise parts of the manuscript are detailed below, they are usually related to points A and B.

English and structure

Overall the structure of the article should be revised to clarify the novelty and hypotheses underlying the study and the differences with previous studies.
In particular, care should be taken to avoid duplicates and verbosity in the main text, while increasing the amount of information present in the captions of the figures which is currently limited.
This issue is especially visible between the Results and Methods, where equations 1-3 and 10-13 are the same.

Various issues:

- Missing hyphens for compound adjectives.
- L280 should read "consistent" and not "coherent".
- Remove double citation of [3] in lines 285-286.

Scientific issues

Introduction

The statement

> Additionally, while providing salient testable predictions, the original MSP growth rules have specifically been developed for excitatory neurites only—they do not provide activity dependent growth rules for inhibitory neurites, nor do they reproduce the experimentally observed outgrowth of inhibitory axons from the LPZ.

is strange since the MSP model does include structural plasticity for inhibitory synapses. See equations 6 and 8 of [33] https://journals.plos.org/ploscompbiol/article?id=10.1371/journal.pcbi.1003259 and the sentence

> We postulate Gaussian-shaped growth curves for the activity- dependent formation and deletion of every type of synaptic element, i.e. excitatory and inhibitory axonal elements A, excitatory dendritic elements Dex and inhibitory dendritic elements Din

Furthermore, though the associated outgrowth of inhibitory axons may not reproduce some observations, the absence of a real convergence in observations of inhibitory plasticity because of their great variability make this point rather weak (see latter comments in Discussion).

Results

A. Stability and growth curves

Paragraph above line 73: $dz/dt = 0$ is not sufficient to provide stability, and the condition for stability depends on the neurons type and incoming/outgoing connections.
Generally, stability in the system is complex and not properly defined nor analyzed. This is clearly visible on Figure 3: where the 500 s window to assess the stability of the AI state seems too short given the typical evolution we see afterwards. I would expect the timescale of changes to be at least 2000 s and I do not think that the simulations shown can properly assess the network's stability with respect to SP.

Tables 1 and 2 are extremely unclear, notably regarding how these facts are verified in simulations (especially as "stability" is not defined). What does the absence of entry for repair in the middle column mean?

Lines 201-204: what does the following sentence mean by "stable state"?

> While a few other pre-synaptic growth curves did allow simulations to show an increase in activity in the LPZ and a loss of activity outside it, the networks in these simulations did not re-balance to a stable state.

Similarly, lines 209-210, what is meant by "stabilize" and "hyperactive"?

> simulations require the growth rates of inhibitory axonal elements to be high enough to stabilize the large number of hyperactive neurons outside the LPZ

What happens if the growth rate is not high enough?

Most of the growth curves chosen on Figure 5 are not stable in themselves but only when combined together or thanks to the network properties; this fact should be analyzed and discussed in more details. For instance, for excitatory neurons, the stable fixed-point of the activity is η, the instability of the z^E_{pre} curve is prevented by the choice of a stable curve for z^E_{post}, and how the instability of z^I_{pre} might be stabilized by through network retroactions is non-trivial, especially given how ψ is chosen (see remark in Methods).
The simple fact that this system may not be stable could, in itself, explain the results shown on Figure 13.

B. Increased synchrony

From the results in Butz et al. [33], one may suspect that this high growth rate and low η^I_{pre} are the reason for the more synchronous behavior that is shown on Figure 4; this should be discussed.

Discussion

258-259: the qualitative changes might be recovered, but not the actual timecourse since the timescales are way shorter.

A. Stability and growth curves

Lines 284-286: If the study by Knott et al [3] finds a proportional increase in the number of inhibitory spines associated to increased stimulation, their observations explicitly states that their is also a significant increase in the number of excitatory spines, this fact goes against the proposed rule for excitatory spines.

Overall, evidence supporting any specific choice of growth curves seems quite limited, which is why (unless stronger numerical evidence is provided) some additional care should

be taken, acknowledging that while the chosen set is reasonable, other combinations or parameters may also lead to similar if not more suitable results.
In particular, this considerably weakens the statement on the loss of the AI state after repair (see remark B below).

Lines 313-315: "Finally, our simulation results indicate that the suggested growth rules, while derived from network simulations, can contribute to the stability of activity in individual neurons (Fig. 10)."
This assertion and the experiment illustrated on Figure 10 are true under the hypothesis of a balanced network, with homogeneous synaptic weights, near its steady-state (which is in itself an interesting result).
Whether SP has the same effect in the situation studied in the manuscript (where the whole network goes away from its previous steady state, individual neurons have synapses with different weights, and the input received in the LPZ are a priori unbalanced and depend on the neuron's own activity and type) or in general is however not a trivial question.
The activity resulting from SP alone and the synchronization obtained in the "successful recoveries" already cast some doubts about this general stabilizing effect, which is why I think the underlying assumptions should be specified.

B. Increased synchrony

266-267: it seems to me that a switch from AI to synchronous would greatly affect the function of the network and may point out at limitations of the model rather than anything else, especially given that this result was expected based on the initial study by Butz et al. [33].
Until a more exhaustive numerical exploration (or analytical analysis) of the parameter space is performed, there is no reason to believe that a recovery maintaining the AI activity is impossible.

Methods

Lines 542-543: This sentence sounds like that the growth curves for excitatory and inhibitory spines are the same ($\eta_I = \eta_E$, $\epsilon_I = \epsilon_E$), which should not be the case according to the choices made.
The evolution shown on Figure 10 suggests that the correct growth curves (shown on Figure 5) were used; the sentence should therefore be clarified to state the difference between the growth curves, with $\eta_I = \epsilon_E = \psi$.

One may actually wonder, though, whether the distinction between excitatory and inhibitory dendritic elements in the model makes sense (since, to the best of my knowledge, this distinction has no biological origin).
It is worth noting that, in the original model, this distinction had much weaker implications since the parameters for both types where identical.

Table 5 : \nu entries should have units

A. Stability and growth curves

Lines 451-452: If I understand correctly, the value of \psi for differs for each neuron in the network.
If so, the choice to assign a given \psi to each neuron based on the activity of that neuron at a precise point in time, due to the STDP, seems rather arbitrary, especially since the STDP and the homeostatic plasticity have separate roots.
Wouldn't it make more sense to choose the same \psi for all inhibitory neurons and another one for the excitatory neurons (e.g. the median or average values)?
I think a justification of this choice and a discussion of its consequences are necessary.

Implementation/technical issues

1. Though the changes made to the NEST kernel are straightforward, they may quickly pose a significant challenge to most users that would want to reproduce the results (as installation methods and platform requirements change) or assess the impact of bugs found in NEST after the fork diverged from the main repository (which was already more than two years ago, in June 2018).
   Furthermore, citation for NEST is "Jordan J, Mørk H, Vennemo SB, Terhorst D, Peyser A, Ippen T, et al. NEST 2.18.0; 2019. Available from: https://doi.org/10.5281/zenodo.2605422", which seems wrong if the code used indeed corresponds to the linked branch.
   I would suggest to merge the NEST 2.20 release into the branch so that this issue is at least be postponed by a couple of years and so that users have an easier way to assess changes and bugs: they would be compared to a release rather than a random commit.
   Similarly, for simulations that do not involve structural changes, this would tell users that they should be able to reproduce them using the 2.20 release.
2. It is unclear how the timestep used to update the structural plasticity (1 second in this

study) may affect the results; I think that a brief investigation of its influence would be necessary, if only as supplementary information, and compared to the timestep used in Butz et al. (100 ms).
