# Supplementary material for: Growth rules for the repair of Asynchronous Irregular neuronal networks after peripheral lesions
Source: PLoS Comput Biol. 2021 Jun 1;17(6):e1008996. doi: 10.1371/journal.pcbi.1008996 (PMC8195387; doi:10.1371/journal.pcbi.1008996)
Supplement: Attachment — Submitted filename: 20201124-SinhaEtAl2020-review-responses.pdf [file pcbi.1008996.s002.pdf]

UH Biocomputation Group,
University of Hertfordshire,
Hatfield AL10 9AB,
UK.
November 24, 2020

The Editors,
PLOS CB.

Dear Madam/Sir,

Thank you very much for your consideration.

We have made the requested modifications to the manuscript to clarify the scope of the work and better present our evidence to support the predictions generated by the modelling study.

We would like to thank the reviewers for their comments and believe that addressing their comments has substantially improved the manuscript.

Please also find attached:

- a revised version of the manuscript,
- a version of the manuscript indicating the changes that we have made.

Reviewer 1

Understanding the mechanisms that enable the brain to develop and then to recover from insults is a very important, but still understudied, topic in neuroscience. Computational modelling has a major role to play in such research by allowing precise investigation of the effects of different "rules" that determine plasticity, both synaptic and structural, in neural networks. This manuscript presents a study that adds significant new aspects to the earlier work of Butz and van Ooyen (MSP model) and provides some interesting predictions that can be the subject of future experimental investigation. The manuscript is generally well written and presented, but could do with clarification of several points:

1. It is unclear whether all the main simulation results presented include all forms of structural plasticity: this would seem to be the case eg. Fig 3 caption just refers to "our structural plasticity mechanism". But the Results section then discusses the post-synaptic changes and the pre-synaptic changes separately, which led me to think you trialled them one at a time (which I do not think you did). This should be clarified in the text.

Thank you very much for your review. In the first section of results, the complete network model of peripheral lesioning and repair is summarised where structural plasticity is active at all neurites and synaptic plasticity is active at the IE synapses. In the following sections, we describe the derivation of the growth rule regimes that allow this simulation of experimentally observed network dynamics one by one. The growth rules were derived sequentially with post-synaptic rules derived first, followed by the pre-synaptic rules. Since the change in connectivity in the network depends on structural plasticity at all neurites, all the figures in the paper are generated from the complete (same) network simulation where structural plasticity is active at all neurites. Only the figures that show single neuron simulations and results where either synaptic or structural plasticity were disabled are generated from other simulations. In these sections this is explicitly noted.

To clarify this, we have updated the caption of Figure 3 to say:

> Structural plasticity is then activated at all neurites—pre-synaptic and post-synaptic, excitatory and inhibitory—to confirm that the network remains in its balanced AI state (panel 1 in A).

In Section 1, we have updated

> Once this AI state is achieved, homeostatic structural plasticity is enabled, and it is confirmed…

to say:

> Once this AI state is achieved, structural plasticity is enabled at all neurites, and it is confirmed…

1. (a) Also, combining tables 1 and 2 would allow for easier comparison of the pre- and post-synaptic element rules.

Thank you. We agree and have combined tables 1 and 2 into one table.

2. Reproducing the time course of recovery is highlighted a number of times (eg line 258 in the Discussion) but it is not made precise what features of the time course are apparent from the simulations.
   (a) Is it mostly the distinction between activity seen just post the lesion time and that arrived at in the steady-state?
   (b) How does simulation time relate to real time in the nervous system: are you using realistic time scales?
   (c) What does experimental data indicate about the time course of axon outgrowth and synapse formation or loss?
   (d) What determines the time course in the model? It would seem that the major determinant is the change in [Ca] level as determined by its decay time constant, and this is applied uniformly to all forms of structural plasticity. Does experimental data indicate different time courses for axonal outgrowth versus synapse formation / loss and could this be accounted for through the use of different time constants for the separate plasticity processes?

Thank you. Yes, as stated in (a), the novel feature of the model is the evolution of the network during the repair process. Our new model correctly simulates the characteristics of the change in network connectivity after peripheral lesion—the restoration of activity to neurons in the LPZ by an ingrowth of excitatory to and the outgrowth of inhibitory axons from it.

Similar to Butz and van Ooyen's work, the structural plasticity mechanism is "fast forwarded" and does not run at biologically realistic time scales. On the computing cluster, the simulation time of 20,000 seconds takes 7 days using 128 computing nodes. Thus, the structural plasticity repair process observed in experiments over a period of months (Keck, Mrsic-Flogel, et al. (2008) and Yamahachi et al. (2009) imaged over a period of 2 months) is compressed to a little over 5 hours in our simulations. It is, therefore, not currently possible to accurately map the rates of the various processes in the model to rates observed in biology and vice-versa.

We have discussed that a future refinement of the growth curves using detailed single neuron multi-compartmental models presents an important next step in adding to our understanding of activity dependent structural plasticity. This would allow for more faithful modelling of the structural plasticity processes, perhaps at biologically realistic time scales. We have added this to the discussion, and also added a paragraph to clearly state the speeding up of structural plasticity mechanisms in the methods. Finally, since "time course" may imply that the simulations closely resemble the exact timing of events observed in experiments to readers, we have changed the phrase to "course" to remove the association with precise timing.

---

3. Clarify in the text that the peri-LPZ is not part of the deafferented zone, but rather is the area referred to when talking about neurons just outside the LPZ. Or if I am mistaken, then definitely clarify what neurons are outside the LPZ and where we can see the increase in activity in these following a lesion (Figures 3 and 4 supposedly illustrate this but only show LPZ-C and peri-LPZ).

Thank you for pointing out that this was not clear. The peri-LPZ is the ring of neurons directly outside the LPZ. We have now listed the four regions in the first results section to clarify which are in and which are outside the LPZ:

> We simulate a peripheral lesion in the network by deafferenting a spatial selection of neurons to form the LPZ. For analysis, following experimental lesion studies, we divide the neuronal population into four regions relative to the LPZ (Figure 1B). The LPZ is divided into two regions:
>
> - LPZ C: the centre of the LPZ (Red in Figure 1B).
> - LPZ B: the inner border of the LPZ (Yellow in Figure 1B).
>
> Neurons outside the LPZ are further divided into two regions:
>
> - P LPZ: peri-LPZ, the outer border of the LPZ (Green in Figure 1B).
> - Other neurons: neurons further away from the LPZ (Grey in Figure 1B).

4. The plots in figures 3 and 4 seem a little counter-intuitive: in figure 3, the firing rates remain somewhat variable in the LPZ throughout, yet there is a marked reduction in variability in the peri-LPZ; whereas in figure 4, the CV of the ISI drops in the LPZ (and how is this calculated if there is no activity in the LPZ following the lesion?), but remains somewhat static in the peri-LPZ?

Thank you for pointing out this apparent discrepancy. The firing rate in Figure 3 is the mean population firing rate that is calculated over a sliding window of 2.5s that advances in steps of 100ms. The temporal evolution of the mean population firing rate at this coarse time scale does not provide any information about the temporal fine structure of the individual spike trains, which is quantified by the ISI CV plot in Figure 4a.

In the absence of spikes the ISI CV cannot be calculated, which explains the discontinuity in the ISI CV of LPZ C neurons in Figure 4a (second dashed line at approximately 2000s). The length of the raster snapshots (1s) is smaller than the window used to calculate the ISI CV (2.5s), thus even though no spikes are visible in panel 3 of Figure 4b (4000 s), spikes in the longer window allowed the calculation of the ISI CV value at this time point (third dashed line at 4000s).

We have clarified the discontinuity in the ISI CV plot in the caption of Figure 4.

4 (a) Also, fig 3A and 4B would both be improved by a clear indication of the neurons inside the LPZ eg circles in 3A and a line covering all the panels in 4B (not just at the y-axis)

Thank you for this suggestion. We have modified Figures 3A and 13A-C to include circles showing the LPZ region and added a note in the captions. We have also added a horizontal line in Figure 4B, and updated the colours used in the raster plots to match the colours used in Figure 4A.

5. Can the increase in firing in the peri-LPZ following the lesion be quantified eg as a percentage of the pre-lesion firing level? It would seem to be more significant in the inhibitory neurons (fig 3C), but maybe it is not, if quantified.

Thank you for suggesting this. We have calculated an increase of approximately $8\,\%$ and $19\,\%$ in E and I neurons in the peri-LPZ region, respectively. We have added this information to the manuscript:

> For neurons outside the LPZ, on the other hand, our simulations show an increase in activity suggesting a net loss of inhibition rather than excitation ($t = 2000\,\mathrm{s}$ in Figure 3C) (~$8\,\%$ and ~$19\,\%$

increases in mean firing rates of E and I neurons in the peri-LPZ in the first 100 seconds after deafferentation, respectively).

6. How, exactly, were the growth rules tuned (eg line 132)? Was it purely done qualitatively on the basis of most neurons returning to a pre-lesion mean firing rate? Was the level of AI quantified in some way and also used as a measure (at least for neurons outside the LPZ) to tune against?

Thank you for pointing out these omissions. Since the structural plasticity mechanism in MSP is based on the time averaged activity in neurons, the growth curves were tuned iteratively over simulations using the "internal" $[Ca^{2+}]$ metric. The structural plasticity mechanism, however, does not take synchronicity into account and so, the AI metric was not used to judge repair in the network. The AI metric was formally defined by Vogels et al. (2011) as:

$$(\text{ISI CV} > 1) \wedge (\sigma_{rate} < 5\,\text{Hz}) \tag{1}$$

where the ISI CV is the mean coefficient of variation of the inter-spike intervals (ISI) of neurons, and σ_{rate} is the standard deviation of the population firing rate. We continue to use their formalism, and have added this definition of the AI state to the manuscript as Equation 17.

7. How significant is the size of the LPZ (relative to the network size) for the time course of recovery and the emergence of the synchronous activity pattern seen in the LPZ?

Thank you for raising this issue. The size of LPZ is a free parameter in the network and its effect on the repair remains a potential topic for future work. Preliminary simulations with a larger LPZ did not show any effect of LPZ size other than requiring more time for recovery. However, we decided to focus on deriving families of growth curves necessary for repair. Thus, we fixed the size of the LPZ, and tuned the growth curves such that the network did successfully repair in this configuration. Simultaneously exploring the effect of growth curves and the size of the LPZ is computationally prohibitive.

8. The Discussion neglects to talk about (lines 288 onwards) the outgrowth of inhibitory axons from the LPZ, experimental evidence for which was cited in the introduction [6], and forms an important outcome of the simulations.

Thank you - we agree that this should indeed be added to the discussion. We have now added the following text to the discussion to address this:

> For inhibitory pre-synaptic elements, where our model correctly reproduces the outgrowth of inhibitory axons from the LPZ as observed in experiments (Marik et al. 2010), we refer to Schuemann et al. who report that enhanced network activity reduced the number of persistent inhibitory boutons (Schuemann et al. 2013) over short periods of time (30 minutes) in organotypic hippocampal slice cultures. However, these experiments also found that prolonged blockade of activity (over seven days) did not affect inhibitory synapses, contrary to the reports from peripheral lesion studies (Chen et al. 2012; Keck, Scheuss, et al. 2011). A prediction from our simulations is that the rates of formation of inhibitory pre-synaptic elements were required to be much greater than that of other neurites to arrest extra excitation in the network. Here, this requirement is borne out of the small proportion of inhibitory neurons that stabilise the activity of the complete neuronal population. This may not be necessary in the brain, however, where activity is stabilised by a multitude of homeostatic mechanisms (Turrigiano 1999).

Reviewer 2

The manuscript addresses possible mechanisms of repair in neuronal networks that are partially deprived of their input, for example as a consequence of peripheral lesions. The scientific scope of the work is very similar to a paper published in PLOS Computational Biology many years ago (Butz & van Ooyen, 2013). The core concept of the solution proposed in the new manuscript – homeostatic structural plasticity – was

in fact brought up in several papers by Arjen van Ooyen and colleagues around the year 2009. The claim of the new manuscript is that the original idea does not work any more in the biologically more realistic setting of asynchronous-irregular (AI) network activity. Imposing additional functional plasticity to all inhibitory synapses in the network, however, is found to mitigate these problems, and successful repair could be demonstrated in numerical simulations.

In view of the strong overlap in concepts and results of the new submission and a previous publication, the question arises, whether the novelty represented by the submitted manuscript deserves a separate publication of it at all. For that reason, I will discuss strengths and weaknesses of the new manuscript in comparison to the previous publication.

Thank you very much for your review.

We would like to note that our manuscript does not suggest that the original idea of homeostatic structural plasticity does not work. Indeed, our post-synaptic growth curves are homeostatic as they maintain the activity of the neuron at its optimal level. Our results show that the particular configuration of growth curves proposed by Butz and van Ooyen (2013) do not permit network repair in the biologically plausible cortical network model by Vogels et al. (2011) that we use in our simulations. Thus, we follow the same method of reproducing characteristics of repair after deafferentation as Butz and van Ooyen (2013) to investigate and suggest new configurations of growth curves required for repair in this network model. This does not contradict previous work. We have made a number of changes to the manuscript to ensure that this is clearly stated in the text.

As pointed out by Reviewers 1 and 3, our work represents the next logical step in refining the growth curves by the inclusion of more biological realism. We believe that our results contribute to the understanding of structural plasticity and merit dissemination to the research community.

1. In their introduction, the authors of the new study make the following statement: “Since the peripheral lesion model proposed by Butz and van Ooyen [33] was not based on a balanced cortical network model with biologically realistic AI activity, their hypothesised growth rules did not elicit repair in our simulations.” This claim provides justification to take up the issue again and propose a new solution to it. The logic of this statement, however, is problematic, as no analysis of sufficient generality was performed in the submitted manuscript. Whereas the authors of the original study used the Izhikevich neuron model, the new study employs an integrate-and-fire neuron model with conductance-based synapses. In both cases, 80% of all neurons were excitatory, and the remaining 20% inhibitory. The original study considered networks comprising 400 neurons in total, whereas the new study seems to have performed simulations of a 25-fold larger network, following Vogels et al. (2011). The actual numbers used are not revealed in the manuscript, however.
   What then is the basis of the claim that the 2013 paper did not consider networks in the AI state? This issue was briefly addressed in Fig. 12 of the original publication, which displays only population activity traces, no spike trains. Although no formal analysis was performed, the visual appearance of the traces shown indeed suggests AI-like activity. Interestingly, the authors of the new paper do not provide any formal analysis of this issue, neither concerning their own networks, nor the networks of the old paper. The activity states of their simulated networks are not characterized quantitatively, and the spike trains shown in Fig. 4 of the submitted manuscript do not allow any conclusions either, they do not even have a proper time axis. Therefore, a convincing quantitative underpinning of the above-mentioned claim is inevitable.

Thank you for pointing out that we should have added a formal definition of the AI regime to our manuscript. We agree.

Since we use the cortical network model developed by Vogels et al. (2011) in our work, we also use the AI

metric that was used by them in their work. They defined the AI state formally as:

$$(\text{ISI CV} > 1) \wedge (\sigma_{rate} < 5\,\text{Hz}) \tag{2}$$

where the ISI CV is the mean coefficient of variation of the inter-spike intervals (ISI) of neurons, and σ_{rate} is the standard deviation of the population firing rate. We have added this definition of the AI state to the manuscript as Equation 17. The raster plots in Figure 4, as stated in the caption, cover a 1 second interval.

Since neither the source code from Butz and van Ooyen (2013), nor the data generated from the work has been made publicly available, and additionally, since the firing characteristics of the network were not discussed in detail, it is difficult to provide objective evidence about the firing regime in the network. We can, therefore, only estimate some related metrics. In Figure 12 A, in the first panel that represents the normal network before deafferentation, there are approximately 10 neurons firing in every 1ms bin shown, for 1000 ms—a total of 10,000 spikes in the 1 second window. These figures include a total of 70 LPZ + 97 peri-LPZ = 167 neurons. Given that the network as a 4:1 ratio of E:I neurons, we approximate that this includes (4/5 of 167) = ˜130 E neurons. The mean population firing rate can thus be inferred to be: $10,000/130 \approx 75\,\text{Hz}$. Spontaneous firing of cortical neurons, however, has been observed at much lower rates ($< 20\,\text{Hz}$ (Evarts 1964; Destexhe and Paré 1999; Hubel 1959; Steriade 1978)).

As noted above, since a complete analysis of the network firing regime in Butz and van Ooyen (2013) cannot be carried out, we have amended the introductory text to read as follows:

> Access to such data and recent advances in simulation technology have enabled computational modelling of activity dependent structural plasticity (Butz, Van Ooyen, and Wörgötter 2009; Deger et al. 2012; Butz and van Ooyen 2013; Butz and van Ooyen 2014; Butz, Steenbuck, and van Ooyen 2014a; Butz, Steenbuck, and van Ooyen 2014b; van Ooyen and Butz 2017). In their seminal work, Butz and van Ooyen introduced the MSP framework (Butz, Van Ooyen, and Wörgötter 2009). They demonstrated its utility by simulating a peripheral lesioning study to explore the activity dependent growth rules of neurites (Butz and van Ooyen 2013; Butz and van Ooyen 2014). Their analysis suggests that the restoration of activity could only be caused by the experimentally noted inward increase in excitatory lateral projections into the LPZ when dendritic elements sprouted at a lower level of activity than their axonal counterparts. Further, since excitatory and inhibitory synaptic elements were treated identically in their model, and this results in inhibitory projections also flowing into the LPZ instead of growing outwards from the LPZ, Butz and van Ooyen also discuss that the contribution of inhibitory neurons to the repair process remains an important open question. A computational model of peripheral lesioning that reproduces all features of the repair process in cortical networks is therefore still lacking.
> Here, as the next step towards improving our understanding of activity dependent structural plasticity in cortical networks, we build on Butz and van Ooyen's work to re-investigate activity dependent growth rules for neurites in the biologically plausible cortical network model developed by Vogels, Sprekeler et al. (Vogels et al. 2011). Unlike in (Butz and van Ooyen 2013) where the cortical network to be deafferented was "grown" using a pre-set free parameter, the Vogels, Sprekeler network model explicitly incorporates cortical network characteristics. Additionally, it is also balanced by homeostatic inhibitory STDP to a low frequency AI (spontaneous) firing regime as observed in the mammalian cortex (Brunel 2000; Destexhe, Rudolph, and Paré 2003) and has been demonstrated to function as an attractor-less store for associative memories (Vogels et al. 2011). By deafferenting this network and reproducing the course of repair as reported in experimental work, we systematically derive activity dependent growth rules for all neurites—excitatory and inhibitory, pre-synaptic and post-synaptic.
> . . .

As already discussed in the manuscript, the growth rules predicted by our work do not conflict with the ones suggested by Butz and van Ooyen. Different networks with different characteristics may exhibit different growth rules:

> Our simulation results do not imply that these are the only configurations of activity dependent growth rules that can underlie the turnover of neurites. Given the variety of neurons and networks in the brain, many configurations (and a variety of growth curves in each configuration) may apply to neurons. The results suggested here are hypothesized using an inhibition-balanced AI cortical network model, and so must be limited to such networks. As an example of a different configuration of growth curves that replicated repair in a different network model, Butz and van Ooyen's simulations proposed that all neurites are sprouted when neurons have less than optimal activity, and that the condition necessary for repair by an ingrowth of excitatory connections is that dendritic elements should be formed before axonal ones (Butz and van Ooyen 2013).
> ...

The numbers of neurons, defined as N_E and N_I, are listed in Table 5 as 8000 and 2000 respectively. We have now also added a reference to Table 5 in the caption of Figure 1.

2. The authors of the new study claim that the hypothesized growth rules did not elicit repair in their simulations. This statement is also very problematic. Maybe the authors just have not tried hard enough to get it to work. Many parameters are different compared to the original setting, so it would not be surprising if also some of the parameters of the plasticity rule need adjustment. The only acceptable argument why it cannot work would be based on a mathematical analysis of the situation, but no such analysis is provided in the manuscript. Instead, the authors suggest an entirely new component of the model (inhibitory STDP) to make network repair possible. This scenario could be accepted as one possible ("sufficient") solution to the problem. In their abstract, however, the authors make the claim that their solution is the only one possible ("necessary"). They claim "Lastly, we observe that our proposed model of homeostatic structural plasticity and the inhibitory synaptic plasticity mechanism that also balances our AI network are both necessary for successful rewiring of the network." No result presented in the manuscript can support this claim. This issue needs clarification.

Thank you for raising this issue. The configurations of growth curves suggested by the model are indeed necessary for repair in our model. This arises from the differential effects of deafferentation on neurons in and outside the LPZ. As shown in the manuscript, whereas the activity of neurons in the LPZ does drop as a result of deafferentation as expected, the activities of neurons outside it *increases*—a net loss of inhibition. Thus, neurons outside the LPZ *must* lose excitatory inputs and gain inhibitory ones to reduce their activity. The growth rules proposed in Butz and van Ooyen (2013) do not allow this. Based on these growth rules, an increase in activity beyond the homeostatic level results in a retraction of all neurites—excitatory or inhibitory, pre-synaptic or post-synaptic. It was this observation that led us to investigate new growth rules. We have clarified the relevant text in the discussion to clearly state that the growth rules proposed here are necessary for repair in this particular inhibition-balanced cortical network setup, but that other growth rules like the ones proposed in Butz and van Ooyen (2013) may apply to other networks.

We agree that in the absence of a complete parameter search, which is not currently tractable due to the high computational costs of these simulations, the particular growth curves used in the paper represent only one possible set of parameters. We have updated the manuscript in multiple locations to stress that the particular configuration of growth rules is necessary, but not the particular set of parameters governing the growth curves used here. We note that this modelling methodology is very much in line with that used in Butz and van Ooyen (2013), where the exact parameters governing the growth curves (η_D, η_A, ϵ) are not derived by a parameter search or mathematical analysis. Instead, perhaps also limited by the computational costs of running a complete parameter search, the required configurations are also obtained there by matching the course of repair in simulations to experimental observations and elimination of other configurations that lead to "aberrant network reorganization". The general issue of a lack of tools for efficient modelling of structural plasticity that will make complete parameter searches tractable has been discussed in our manuscript.

Our simulations show that after deafferentation, in the presence of only structural plasticity, the network does undergo rewiring to return activity to deprived neurons in the LPZ. However, in our model, activity does not re-balance to a low firing AI state if synaptic plasticity is not present. As documented, this indicates

that the larger, discrete changes made by structural plasticity to synaptic conductances are insufficient to balance excitation and inhibition in the network. The much smaller tweaks made by synaptic plasticity are necessary to finely tune inhibitory conductances to achieve this balance. The claim in the abstract is, therefore, supported by our results in the scope of this modelling study.

3. The new solution proposed in the manuscript involves two different types of synaptic plasticity, homeostatic structural plasticity and inhibitory spike-timing dependent plasticity. Whereas the latter is relatively fast with time scales in the range of tens of milliseconds, the former is rather slow, operating on time scales that are several orders of magnitude larger. But how then can fast inhibitory plasticity compensate for the deficits of slow homeostatic plasticity? On page 9, second to last paragraph the authors state: "Simulations require the growth rates of inhibitory axonal elements to be high enough to stabilise the large number of hyperactive neurons outside the LPZ." It remains unclear why inhibitory plasticity cannot compensate for this. Generally, the new manuscript does not provide sufficient insight into the question how the two types of plasticity interact. For example, a simple tracking of inhibitory amplitudes in the course of time, together with structural changes that happen simultaneously, would shed light on this question. Such extra insight is absolutely necessary to justify a solution that is considerably more complicated than previous suggestions.

Thank you for this comment. As discussed above, the model deals with two types of plasticity, one synaptic and one structural. Synaptic plasticity was originally included as it is a feature of the cortical network model by Vogels et al. (2011) - it can only modulate the strengths of already existing synapses, but it cannot create or remove synapses. The restoration of excitation to the deprived neurons in the LPZ requires the formation of new synapses, which the structural plasticity mechanism creates. Similarly for inhibition, the inhibitory STDP mechanism only modulates efficacies of synapses that already exist. The structural plasticity mechanism is necessary to create new inhibitory synapses to inhibit neurons. Once these new inhibitory synapses have been created, the inhibitory STDP mechanism is able to modulate them to balance both local and global excitation with inhibition. Thus, while the two types of plasticity modify the network at different temporal and spatial scales, they both contribute to the balancing of excitation and inhibition in the network.

4. The authors of the submitted manuscript discuss different options for the growth curves describing the homeostatic controller. However, they discuss only growth curves that arise from a translation of the Gaussian curve in x and y direction. What is the exact motivation of this somewhat restricted perspective? As the most important parameter is the slope of the curve in the set-point, other transformations (e.g. a stretching) of the growth curves actually seem more relevant. Why does one need a Gaussian shape for the axonal elements to begin with? Wouldn't the creation of axonal elements at a constant rate provide a simpler (better) solution? A more systematic account of these questions is necessary to go beyond the insight from previous studies.

Thank you for this comment. The use of Gaussian growth curves is a feature of the MSP framework. The work presented here makes use of the MSP framework to investigate structural plasticity. Investigations of other families of growth curves beyond the MPS is, unfortunately, beyond the scope of our work, given the computational costs associated with it.

The necessary condition that we apply to derive the growth curves is that at the set-point (the optimal activity of the neuron, ψ), no change in neurites should occur in neurons ($dz/dt = 0$ for all neurites). By reproducing the course of repair from experiments, we are able to ascertain which of the two fixed points of each Gaussian growth curve, where $dz/dt = 0$ holds, should take the value of ψ. As discussed above, a systematic variation of the parameters of the growth curves (which would stretch or squeeze them in x and y directions) is intractable with the current research technologies and so, we limit our results to the prediction of configurations of the activity dependent growth curves only. The exact parameters for growth curves, which may vary for different cell types, remains to be ascertained.

Furthermore, our preliminary simulations showed that constant availability of axonal contacts would not reproduce the characteristics of the course of repair observed in experiments. If excitatory axonal points are

available to the deprived neurons in the LPZ from themselves, since synapse formation is distance dependent and more likely between neurons closer together, they will be prevented from forming synapses with free axonal neurites on neurons outside the LPZ that are further away—no longer reproducing the inward sprouting of excitatory axons to the LPZ. Similarly, if inhibitory axons are always being formed, neurons outside the LPZ that have more activity than necessary will form inhibitory synapses with other nearby neurons outside the LPZ instead of the more distant neurons in the LPZ—no longer reproducing the outgrowth of inhibitory axons from the LPZ.

In general, we attempted to remain as faithful to Butz and van Ooyen's MSP as possible to limit the scope of the work to the derivation of activity dependent dynamics that allow repair in the model following the course observed in experiments.

Reviewer 3

The present study proposes a simple yet qualitatively significant change to the MSP model, proposed by Butz et al, that provides a generic framework to describe post-lesion repair. They apply this framework to peripheral lesioning of a balanced AI network aimed at modeling cortical activity. In addition to the change in the MSP model, they also include I$\rightarrow$E STDP plasticity and strength-dependent deletion of synapses to their simulation, then assess numerically how both plasticity mechanisms must work together to generate a partial recovery. The rationale behind the evolution of post- and pre-synaptic structures for both collective and single-cell dynamics also seems clear and consistent, even though limited experimental and numerical evidence is provided as to why other possibilities should not be considered. Required code to reproduce the result is provided using free open-source licenses.

I believe that this work represents an interesting contribution and a useful, logical advancement for studies on structural plasticity. I would support its publication in PLOS CB provided one major question is answered and some restructuring is performed on the manuscript, including the addition of precision/discussions (notably a more in-depth comparison with previous studies).

The one major issue that I have with this study concerns the stability of the network activity with respect to the rules chosen for structural plasticity (SP). Indeed, the authors only looked at the influence of structural plasticity for 500 s in the control state. This seems too short compared to the typical timescales over which the changes are occurring during the recovery (several thousand seconds). Furthermore, most growth curves are intrinsically unstable on their own, making the reason for the overall stability hard to understand, especially as it is not discussed at all (though, to be fair, the non-trivial case of inhibitory neurons was also ignored by Butz et al. in the original model [33]).

Thank you very much for your review. We apologise that the issue of stability was not clear enough in the manuscript. The following paragraph provides a brief summary of the features of the model that ensure stability and outlines the changes to the manuscript.

When the neuron's activity is ψ, since $dz/dt = 0$ for all neurites, no turnover of neurites will occur. Each growth curve has two fixed points where $dz/dt = 0$. The activity of the neuron depends on the inputs it receives through the excitatory and inhibitory synapses formed by its post-synaptic neurites. The growth curves for the post-synaptic elements are placed such that they intersect at the optimal activity level of the neuron: $[Ca^{2+}] = \psi$—this point of intersection acts as the stable fixed points for the two growth curves and the "global" stable fixed point for the neuron. Any deviations in the activity of the neuron will cause changes in the post-synaptic neurites that attempt to return the activity level back to the optimal level. Other configurations of the post-synaptic growth curves can not give rise to these stable fixed points. Further, to ensure that the experimentally observed ingrowth of excitatory connections to the LPZ and outgrowth of inhibitory connections from the LPZ occur during repair, the post-synaptic growth curves *must* be on either side of the optimal activity level.

To clarify this, we have now improved Figure 5 by adding arrows to indicate the optimal activity ψ as the stable fixed point for both growth curves, and updated the figure caption to explain how the post-synaptic growth curves counter any changes in the activity of the neuron away from the optimal level.

Besides this major issue, I feel that there is a need for more thorough justification of several modeling choices and their consequences:

- Are the choices related to the growth curves really ensuring the stability of the activity?

Thank you for the comment. Yes, as documented above, the post-synaptic growth rules impose a homeostatic mechanism at individual neurons. In combination with the inhibitory STDP mechanism, they continuously balance excitation and inhibition for the neurons to maintain their activities at the set optimal point.

- Was the final increase in synchrony not expected given the chosen parameters?

Thank you for this interesting question. The chosen parameters themselves do not provide any intuition on the firing characteristics of the network. They only ensure that the time averaged activity, in terms of the $[Ca^{2+}]$, is maintained at the set level for the neuron.

It has recently been pointed out to us that some work exploring the connectivity of neuronal networks would suggest that the synchrony most likely arises from the increased excitatory coupling in the neurons of the LPZ, as a result of the substantial increase in lateral excitatory connectivity by the structural plasticity process during network repair. We have included this information in the discussion and cited the relevant texts:

> Secondly, our model suggests that while the network may restore its mean activity, the temporal fine structure of the activity, and in particular its AI firing characteristic are permanently disturbed by deafferentation. This change in firing patterns of the network also merits experimental validation, especially given its implications for network function. Given that the inhibitory STDP mechanism is unable to maintain the network in its AI regime following repair by structural plasticity, the deviation from the AI firing regime is likely caused by the alteration of network connectivity during the repair process. Indeed, as Figure 6 shows, neurons in the LPZ C region gain a significant number of lateral excitatory connections from neurons outside the LPZ (3×10^4 before deafferentation at $t = 2000s$ vs 5×10^4 at the end of the repair process at $t = 18000s$), greatly increasing their excitatory input connectivity. This is in line with previous work that indicates that synchronisation may occur in networks of excitatory and inhibitory neurons when the number of inputs received by neurons is more than a critical value (Börgers and Kopell 2003; Brunel 2000; Qu et al. 2013; Golomb and Hansel 2000; Nowotny and Huerta 2003; Papadopoulou et al. 2011). The precise relationship between network sparsity and population firing dynamics in a network balanced by the inhibitory STDP mechanism used here, however, does not appear to have been ascertained yet.

- Why was STDP limited to I→E and instead of (for instance) scaling of excitatory synapses?

The inhibitory STDP mechanism is a feature of the Vogels-Sprekeler cortical network model (Vogels et al. 2011) that represents the normal functioning network and that forms the basis of our work. In their work, Vogels et al. (2011) have shown that this inhibitory STDP mechanism is able to maintain the network in a balanced AI state.

We have improved the introduction to make this clearer:

> Here, as the next step towards improving our understanding of activity dependent structural plasticity in cortical networks, we build on Butz and van Ooyen's work to re-investigate activity dependent growth rules for neurites in the biologically plausible cortical network model developed by Vogels, Sprekeler et al. (Vogels et al. 2011). Unlike in (Butz and van Ooyen 2013) where the cortical network to be deafferented was "grown" using a pre-set free parameter, the Vogels, Sprekeler network model explicitly incorporates cortical network characteristics. Additionally, it is also balanced by homeostatic inhibitory STDP to a low frequency AI (spontaneous) firing regime as

> observed in the mammalian cortex (Brunel 2000; Destexhe, Rudolph, and Paré 2003) and has been demonstrated to function as an attractor-less store for associative memories (Vogels et al. 2011). By deafferenting this network and reproducing the course of repair as reported in experimental work, we systematically derive activity dependent growth rules for all neurites—excitatory and inhibitory, pre-synaptic and post-synaptic.
> ...

- Structural changes are very fast compared to the biological values mentioned in the MSP paper [33] (72 days over the whole timecourse) and the experimental studies that are cited [6, 9] (several days). The necessity of speeding up simulations is understandable (it is also argued in [33]) and it is probably sufficient for the timescales of neuronal activity/STDP and SP to occur on "sufficiently different" timescales to achieve "reasonable modeling". However that "sufficient difference" should certainly be discussed if not proven, especially given the fast growth of inhibitory axons in the current study.

Thank you for raising this issue. The rate of structural plasticity in the simulations is primarily limited by the rate at which connection updates are made—there may be free neurites ready to form new synapses in the network, but these will only be formed at each connectivity update. The synaptic plasticity mechanism acts at millisecond time scales, with a time constant τ_{STDP} of 20 ms. The structural plasticity process, is thus, slow enough. We have amended the Methods section to clarify this:

> The numbers of synaptic elements are updated at every simulator integration time step internally in NEST. Connectivity updates to the network, however, require updates to internal NEST data structures and can only be made when the simulation is paused and incur considerable computational costs. Given that the synaptic plasticity mechanism acts at the time scale of milliseconds ($\tau_{STDP} = 20$ ms), we make connectivity updates at 1 s intervals to keep structural plasticity updates faster than that in biology, but still sufficiently slower than the synaptic plasticity mechanism.

More specific remarks and questions regarding precise parts of the manuscript are detailed below, they are usually related to points A and B.

English and structure

Overall the structure of the article should be revised to clarify the novelty and hypotheses underlying the study and the differences with previous studies. In particular, care should be taken to avoid duplicates and verbosity in the main text, while increasing the amount of information present in the captions of the figures which is currently limited. This issue is especially visible between the Results and Methods, where equations 1–3 and 10–13 are the same.

Thank you for this comment. We have removed the repeated equations and made a number of changes to the captions to improve their information content.

Various issues:

- Missing hyphens for compound adjectives.

Thank you. We have checked the manuscript to ensure the consistent usage of hyphens.

- L280 should read "consistent" and not "coherent".

Thank you for spotting the mistake. We have made this change.

- Remove double citation of [3] in lines 285–286.

Thank you for spotting this error. We have removed the duplicate citation.

Scientific issues

Introduction

The statement

> Additionally, while providing salient testable predictions, the original MSP growth rules have specifically been developed for excitatory neurites only—they do not provide activity dependent growth rules for inhibitory neurites, nor do they reproduce the experimentally observed outgrowth of inhibitory axons from the LPZ.

is strange since the MSP model does include structural plasticity for inhibitory synapses. See equations 6 and 8 of [33] https://journals.plos.org/ploscompbiol/article?id =10.1371/journal.pcbi.1003259 and the sentence

> We postulate Gaussian-shaped growth curves for the activity- dependent formation and deletion of every type of synaptic element, i.e. excitatory and inhibitory axonal elements A, excitatory dendritic elements Dex and inhibitory dendritic elements Din

Furthermore, though the associated outgrowth of inhibitory axons may not reproduce some observations, the absence of a real convergence in observations of inhibitory plasticity because of their great variability make this point rather weak (see latter comments in Discussion).

Thanks for pointing this out. We agree that the statement above did not clearly convey our intended meaning. We have rephrased the paragraph in the introduction to clearly contrast our work and the previous work.

Results

A. Stability and growth curves

Paragraph above line 73: dz/dt = 0 is not sufficient to provide stability, and the condition for stability depends on the neuron's type and incoming/outgoing connections. Generally, stability in the system is complex and not properly defined nor analyzed. This is clearly visible on Figure 3: where the 500 s window to assess the stability of the AI state seems too short given the typical evolution we see afterwards. I would expect the timescale of changes to be at least 2000 s and I do not think that the simulations shown can properly assess the network's stability with respect to SP.

Thank you for this comment. As noted above, for the post-synaptic growth curves hypothesized in the work, $dz/dt = 0$ at $[Ca^{2+}] = \psi$ does indeed act as a stable fixed point. In our model, the growth curves for the post-synaptic elements are placed such that they intersect at the optimal activity level of the neuron. Governed by the post-synaptic growth rules, a neuron will not retract or form new post-synaptic neurites, thus keeping its activity at the required optimal level. Similarly, when the neuron's activity is at ψ, the neuron will also not sprout or retract pre-synaptic neurites.

We ran a longer (˜16000 seconds) simulation where synaptic and structural plasticity are both active, but no deafferentation occurs. Figure 1 below shows that network activity is maintained at the required level.

Tables 1 and 2 are extremely unclear, notably regarding how these facts are verified in simulations (especially as "stability" is not defined). What does the absence of entry for repair in the middle column mean?

Thank you for pointing out that the missing entries are confusing. Tables 1 and 2 are intended to provide a summary of our simulation results that are explained in the text. The missing entry was meant to imply "NA" (not applicable) - if the growth curves do not result in a stable system in the absence of perturbation (deafferentation), they were not considered for simulations with deafferentation at all. We have added "NA" in these missing spaces now to clarify this.

Lines 201–204: what does the following sentence mean by "stable state"? "While a few other pre-synaptic

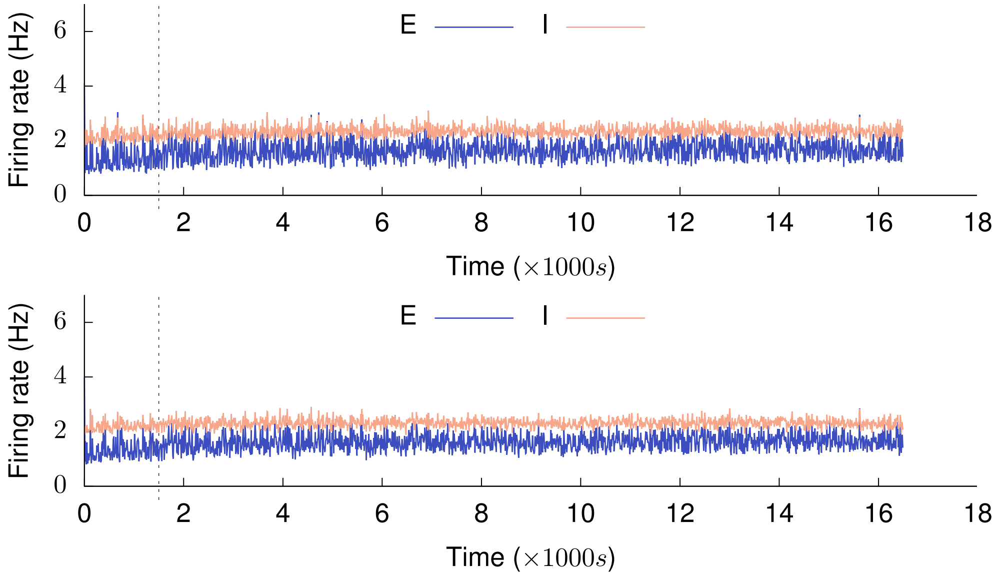

Figure 1: Mean firing rate of excitatory and inhibitory neurons in the LPZ C region (Top) and Peri-LPZ region (Bottom). The synaptic plasticity mechanism is active throughout the simulation, but the structural plasticity mechanism is activated only after the network has initially achieved its AI firing state, at $t = 1500$ s.

growth curves did allow simulations to show an increase in activity in the LPZ and a loss of activity outside it, the networks in these simulations did not re-balance to a stable state."

Thank you. In the simulations, and so in the paper, the network being in a "stable state" implies it remaining in a regime where the activity of the neurons remains at the optimal level set for each neuron.

Thus, this statement means that other choices for pre-synaptic growth rules did help the network gain activity in the LPZ and lose activity outside it, but did not return the network to its balanced state.

Similarly, lines 209–210, what is meant by "stabilize" and "hyperactive"?

"simulations require the growth rates of inhibitory axonal elements to be high enough to stabilize the large number of hyperactive neurons outside the LPZ"

What happens if the growth rate is not high enough?

Thank you for pointing out that this was not clear. "Hyperactive" here means that these neurons have more activity than their optimal levels. We have replaced the term for clarity.

If the growth rate of pre-synaptic neurites on inhibitory neurons is not high enough, neurons outside the LPZ that have more activity than their optimal level will continue to sprout inhibitory post-synaptic elements and retract excitatory post-synaptic elements. However, since there are no free pre-synaptic inhibitory neurites to form inhibitory synapses with, the inhibitory post-synaptic elements will remain unconnected—unable to increase inhibitory input received by the neuron to reduce its activity. The reduction of activity in these neurons will therefore be dominated by the loss of excitatory inputs, which in turn will result in the disconnection of excitatory synapses in the network. We have noted this in the paper:

> If the growth rate of inhibitory pre-synaptic elements is not high enough, newly sprouted inhibitory post-synaptic elements on these neurons will remain unconnected. Without the additional inhibition, these neurons will rely solely on the loss of excitatory synapses by the retraction of their excitatory post-synaptic elements to reduce their activity back to optimal levels—adversely affecting network function.

Most of the growth curves chosen on Figure 5 are not stable in themselves but only when combined together or thanks to the network properties; this fact should be analyzed and discussed in more details. For instance, for excitatory neurons, the stable fixed-point of the activity is η, the instability of the z^E_{pre} curve is prevented by the choice of a stable curve for z^E_{post}, and how the instability of z^I_{pre} might be stabilized by through network retroactions is non-trivial, especially given how ψ is chosen (see remark in Methods).

The simple fact that this system may not be stable could, in itself, explain the results shown on Figure 13.

Thank you. Yes, as discussed above, the post-synaptic growth curves keep the activity of the neuron at the necessary level, which keeps the growth of pre-synaptic neurites also in check. Figure 13 C, where only structural plasticity acts on the system, is explained by the discrete nature of the structural plasticity mechanism. It can only make relatively large, sudden changes to network conductances, making it harder for the balancing of excitation and inhibition in the network. Whereas the structural plasticity mechanism may bring the network in the neighbourhood of a balanced state, inhibitory synaptic plasticity is required in the model to fine tune inhibition to converge the network to a balanced state.

We have improved both result sections pertaining to the derivation of the growth curves to clarify this. In the section pertaining to the post-synaptic growth curves, we have added:

> Since ϵ^E_{post} and η^I_{post} individually represent the stable fixed points for the excitatory and inhibitory post-synaptic growth curves respectively, the intersection of these growth curves with each other at the neuron's optimal activity level, ψ, ensures that deviations to the neuron's activity away from ψ are countered by the turnover of the neuron's post-synaptic elements. ...

We have edited the section pertaining to the pre-synaptic growth curves to say:

> As summarised in Table 4, only the derived configuration for pre-synaptic growth curves reproduced all experimentally reported features of the repair process: inhibitory axons sprout when neuronal activity is less than optimal, but excitatory axons sprout when activity is more than required. Since the activity of neurons is continuously stabilised by the previously derived post-synaptic growth curves, the turnover of pre-synaptic elements is also kept in check. Thus, as neurons in the network achieve their optimal activity levels, the turnover of both post- and pre-synaptic elements ceases.

Increased synchrony

From the results in Butz et al. [33], one may suspect that this high growth rate and low η^I_{pre} are the reason for the more synchronous behavior that is shown on Figure 4; this should be discussed.

Thank you for pointing out this omission. We have added a brief discussion of the synchrony of the network to the manuscript.

Discussion

258–259: the qualitative changes might be recovered, but not the actual timecourse since the timescales are way shorter.

Thank you. We agree and have replaced "time course" with "course".

A. Stability and growth curves

Lines 284–286: If the study by Knott et al [3] finds a proportional increase in the number of inhibitory spines associated to increased stimulation, their observations explicitly states that their is also a significant increase in the number of excitatory spines, this fact goes against the proposed rule for excitatory spines.

Thank you for pointing out this inconsistency. Knott et al. (2002) report an increase in both excitatory and inhibitory *synapses*, on "double synapses". On re-examining the methods used in their work, we observe

that spines themselves were not directly classified as excitatory or inhibitory. They used GABA immunohistochemistry to classify pre-synaptic neurites as GABA positive or negative. Synapses with GABA positive pre-synaptic elements were classified as inhibitory synapses, and those with GABA negative ones as excitatory.

This, along with the long 24 hour period of stimulation, which may represent multiple cycles of neurite/synapse formation and removal, makes it difficult to draw conclusions from the work on the instantaneous responses of neurons to change in activity described by the growth curves. We have therefore omitted this citation from the paper.

Overall, evidence supporting any specific choice of growth curves seems quite limited, which is why (unless stronger numerical evidence is provided) some additional care should be taken, acknowledging that while the chosen set is reasonable, other combinations or parameters may also lead to similar if not more suitable results. In particular, this considerably weakens the statement on the loss of the AI state after repair (see remark B below).

Thank you for this comment. We agree that experiments and further modelling studies using more detailed biophysical components are necessary and logical next steps to our work. To clarify that the parameters governing the postulated growth curves are not the only possible ones, we have updated the manuscript in several locations to stress that the simulations, and reproduction of the course of repair suggests these configurations for the growth rules in relation to the optimal activity of neurons, and that the curves used in the simulation illustrate these configurations. For example, we have amended the discussion to:

> Our simulation results do not imply that these are the only configurations of activity dependent growth rules that can underlie the turnover of neurites. Given the variety of neurons and networks in the brain, many configurations (and a variety of growth curves in each configuration) may apply to neurons. The results suggested here are hypothesized using an inhibition-balanced AI cortical network model, and so must be limited to such networks. As an example of a different configuration of growth curves that replicated repair in a different network model, Butz and van Ooyen's simulations proposed that all neurites are sprouted when neurons have less than optimal activity, and that the condition necessary for repair by an ingrowth of excitatory connections is that dendritic elements should be formed before axonal ones (Butz and van Ooyen 2013).

The loss of synchrony has been addressed above.

Lines 313–315: "Finally, our simulation results indicate that the suggested growth rules, while derived from network simulations, can contribute to the stability of activity in individual neurons (Fig. 10)." This assertion and the experiment illustrated on Figure 10 are true under the hypothesis of a balanced network, with homogeneous synaptic weights, near its steady-state (which is in itself an interesting result). Whether SP has the same effect in the situation studied in the manuscript (where the whole network goes away from its previous steady state, individual neurons have synapses with different weights, and the input received in the LPZ are a priori unbalanced and depend on the neuron's own activity and type) or in general is however not a trivial question. The activity resulting from SP alone and the synchronization obtained in the "successful recoveries" already cast some doubts about this general stabilizing effect, which is why I think the underlying assumptions should be specified.

Thank you for the comment. The simulation shown in Figure 10 involves an isolated single neuron that is taken out of the context of the network, so the assumption of the presence of a balanced network is not necessary, and we are only investigating the contribution of post-synaptic growth curves. The only assumption relating to the pre-synaptic side is an unlimited availability of pre-synaptic elements.

We have now modified the sentence to say "… suggested post-synaptic growth rules, while derived from network simulations, can contribute to the stability of activity in an isolated individual neuron (Fig. 10)" to explicitly point out that this particular simulation only applies to the post-synaptic rules.

B. Increased synchrony

266–267: it seems to me that a switch from AI to synchronous would greatly affect the function of the network and may point out at limitations of the model rather than anything else, especially given that this result was expected based on the initial study by Butz et al. [33].

Until a more exhaustive numerical exploration (or analytical analysis) of the parameter space is performed, there is no reason to believe that a recovery maintaining the AI activity is impossible.

Thank you. As noted above, the synchrony observed in the neurons of the LPZ is likely caused by the increased lateral excitatory coupling in the neurons by the ingrowth of excitatory connections from the neurons outside the LPZ. This phenomenon remains to be investigated both experimentally and in future modelling work. The manuscript has been updated to include this discussion:

> Secondly, our model suggests that while the network may restore its mean activity, the temporal fine structure of the activity, and in particular its AI firing characteristic are permanently disturbed by deafferentation. This change in firing patterns of the network also merits experimental validation, especially given its implications for network function. Given that the inhibitory STDP mechanism is unable to maintain the network in its AI regime following repair by structural plasticity, the deviation from the AI firing regime is likely caused by the alteration of network connectivity during the repair process. Indeed, as Figure 6 shows, neurons in the LPZ C region gain a significant number of lateral excitatory connections from neurons outside the LPZ (3×10^4 before deafferentation at $t = 2000s$ vs 5×10^4 at the end of the repair process at $t = 18000s$), greatly increasing their excitatory input connectivity. This is in line with previous work that indicates that synchronisation may occur in networks of excitatory and inhibitory neurons when the number of inputs received by neurons is more than a critical value (Börgers and Kopell 2003; Brunel 2000; Qu et al. 2013; Golomb and Hansel 2000; Nowotny and Huerta 2003; Papadopoulou et al. 2011). The precise relationship between network sparsity and population firing dynamics in a network balanced by the inhibitory STDP mechanism used here, however, does not appear to have been ascertained yet.

Methods

Lines 542–543: This sentence sounds like that the growth curves for excitatory and inhibitory spines are the same ($\eta_I = \eta_E, \epsilon_I = \epsilon_E$), which should not be the case according to the choices made. The evolution shown on Figure 10 suggests that the correct growth curves (shown on Figure 5) were used; the sentence should therefore be clarified to state the difference between the growth curves, with $\eta_I = \epsilon_E = \psi$.

Thank you for the comment. Initially, when the neuron is being initialised to resemble a neuron in the network, the same post-synaptic growth rules are indeed used here. With $\nu^E_{post} = 4\nu^I_{post}$, we obtain approximately $z^E_{post} = 4z^I_{post}$ post synaptic neurites to mimic the initial indegree of neurons in the network simulations. Once the neuron has been initialised to this state, the growth curves obtained by our simulations are applied to study the change in input conductance due to fluctuations in the $[Ca^{2+}]$ of the neuron. We have rewritten the Methods section on the single neuron simulations to clarify this:

> The neuron is initialised to a steady state where it exhibits an in-degree similar to neurons in the network simulations when in their AI state. To do so, a constant baseline input current I_{ext} is supplied to the neuron to provide it with activity. The $[Ca^{2+}]$ obtained by the neuron at this time is assumed as its optimal level, ψ. Using identical values of η and ϵ but different ν values for excitatory and inhibitory post-synaptic elements ($\nu^E_{post} = 4\nu^I_{post}$ to mimic the initial in-degree of neurons in our network simulations), and an input current that deviates the activity of the neuron off its optimal level ($< I_{ext}$), the neuron is made to sprout z^E_{post}, z^I_{post} excitatory and inhibitory post-synaptic elements respectively ($z^E_{post} = 4z^I_{post}$). At this stage, the neuron has been initialised to resemble one in network simulations in its balanced state before deafferentation. The current input is returned to its baseline value, thus returning the $[Ca^{2+}]$ to its optimal value, ψ.
> Next, the growth curves for the neuron are restored as per our activity dependent structural plas-

ticity hypotheses to verify that the neuron does not undergo any structural changes at its optimal activity level. ...

One may actually wonder, though, whether the distinction between excitatory and inhibitory dendritic elements in the model makes sense (since, to the best of my knowledge, this distinction has no biological origin). It is worth noting that, in the original model, this distinction had much weaker implications since the parameters for both types where identical.

Thank you for this comment. There is sufficient difference in excitatory and inhibitory synapses in biology to merit their participating neurites to be treated as independent units. For example, while excitatory synapses are generally formed on dendritic spines, inhibitory synapses tend to be formed directly on the dendritic shaft (Wierenga, Becker, and Bonhoeffer 2008). Whereas the model abstracts over these two different categories to refer to both as neurites in the morphology-less point neurons, we believe their treatment as independent units is warranted.

Additionally, as our simulations show, their growth rules must be placed on either side of the optimal activity level for repair to occur in our cortical network model.

Table 5: ν entries should have units

Thank you. We have suffixed the entries in the tables with "per dt".

A. Stability and growth curves

Lines 451–452: If I understand correctly, the value of ψ for differs for each neuron in the network.

If so, the choice to assign a given ψ to each neuron based on the activity of that neuron at a precise point in time, due to the STDP, seems rather arbitrary, especially since the STDP and the homeostatic plasticity have separate roots.

Wouldn't it make more sense to choose the same ψ for all inhibitory neurons and another one for the excitatory neurons (e.g. the median or average values)? I think a justification of this choice and a discussion of its consequences are necessary.

Thank you for the suggestion. Since the activities of neurons of each type, excitatory and inhibitory, are similar when the network is in its AI state, the optimal activities (their time averaged activity) set for them will also be similar. Since the network is in the AI firing regime where neurons have similar but not identical activities, it follows that their optimal activities are also similar but not identical. Furthermore, the variability of our model neurons mirrors the variability of neurons that is found in biology. We have added the following text to the methods section to note this:

> The optimal activity of each neuron, ψ, is set to the activity achieved by the neuron at this point, and its growth curves are initialised in relation to it. Since neurons in the AI network have similar but not identical activities, it follows that their optimal activities are also similar but not necessarily identical. This ensures that the structural plasticity mechanism attempts to stabilise all neurons to the activities they achieved when the network is balanced by the inhibitory STDP to its normal AI state.

Implementation issues

- Though the changes made to the NEST kernel are straightforward, they may quickly pose a significant challenge to most users that would want to reproduce the results (as installation methods and platform requirements change) or assess the impact of bugs found in NEST after the fork diverged from the main repository (which was already more than two years ago, in June 2018). Furthermore, citation for NEST is "Jordan J, Mørk H, Vennemo SB, Terhorst D, Peyser A, Ippen T, et al. NEST 2.18.0; 2019. Available from: https://doi.org/ 10.2281/zenodo.2605422", which seems wrong if the code used indeed corresponds to the linked branch.

I would suggest to merge the NEST 2.20 release into the branch so that this issue is at least be postponed by a couple of years and so that users have an easier way to assess changes and bugs: they would be compared to a release rather than a random commit. Similarly, for simulations that do not involve structural changes, this would tell users that they should be able to reproduce them using the 2.20 release.

Thank you for pointing this out. We have updated our fork of the NEST simulator such that it is based on the on the nearest release: NEST 2.18.0, and ensured that only this release is cited in the manuscript. The simulations that do not use structural plasticity can be run on any NEST release after the 2.12.0 release when the one of the authors contributed the Vogels-Sprekeler model to NEST. We have also noted this in the methods section:

> The Vogels-Sprekeler STDP model was contributed to the NEST simulator in version 2.12.0 (Kunkel et al. 2017). Simulations without structural plasticity can, therefore, be run on any of the newer releases.

- It is unclear how the time step used to update the structural plasticity (1 second in this study) may affect the results; I think that a brief investigation of its influence would be necessary, if only as supplementary information, and compared to the time step used in Butz et al. (100 ms).

Thank you. As discussed above, given the short timescales of synaptic plasticity, the connectivity updates are "sufficiently slow". The connectivity update interval can be reduced as long as it does not interfere with the synaptic plasticity mechanism. We did previously run test simulations to test out the effect of reduced connection update interval. The primary effect is the expected smoother change in the number of neurites, since fewer neurites are formed or removed in the smaller interval but these changes occur more frequently. Additionally, the increased frequency of connection updates also allows the synaptic plasticity mechanism to balance the network in its new configuration more frequently.

Unfortunately, it is not currently tractable to run the complete 18000 second simulation with a smaller connectivity update interval in the computational resources available to us. Reducing the update interval by half doubles the number of times the simulation must be paused, greatly increasing the computing cost. We can show this with shorter simulations. In these simulations, the network runs for the first 1500 seconds to reach its AI state, and then runs for a further 2500 seconds with structural plasticity also enabled (a total of 4000 seconds) (Fig. 2). All simulations are run on the UH high performance cluster using 128 identical cores and are initialised using the same seeds. The time taken for the simulations to complete is:

- ˜22 hours for a connectivity update interval of 1 second,
- ˜37 hours for a connectivity update interval of 0.5 seconds,
- ˜117 hours for a connectivity update interval of 0.25 seconds.

Since there is no deafferentation or repair in these test simulations, however, a very small number of connections are removed or formed. A simulation with deafferentation and repair where more connections are formed and removed would further exacerbate the issue. As can be seen from the mean firing rate plots, there is no qualitative difference in the behaviour of the network.

Yours sincerely,

Ankur Sinha and Volker Steuber

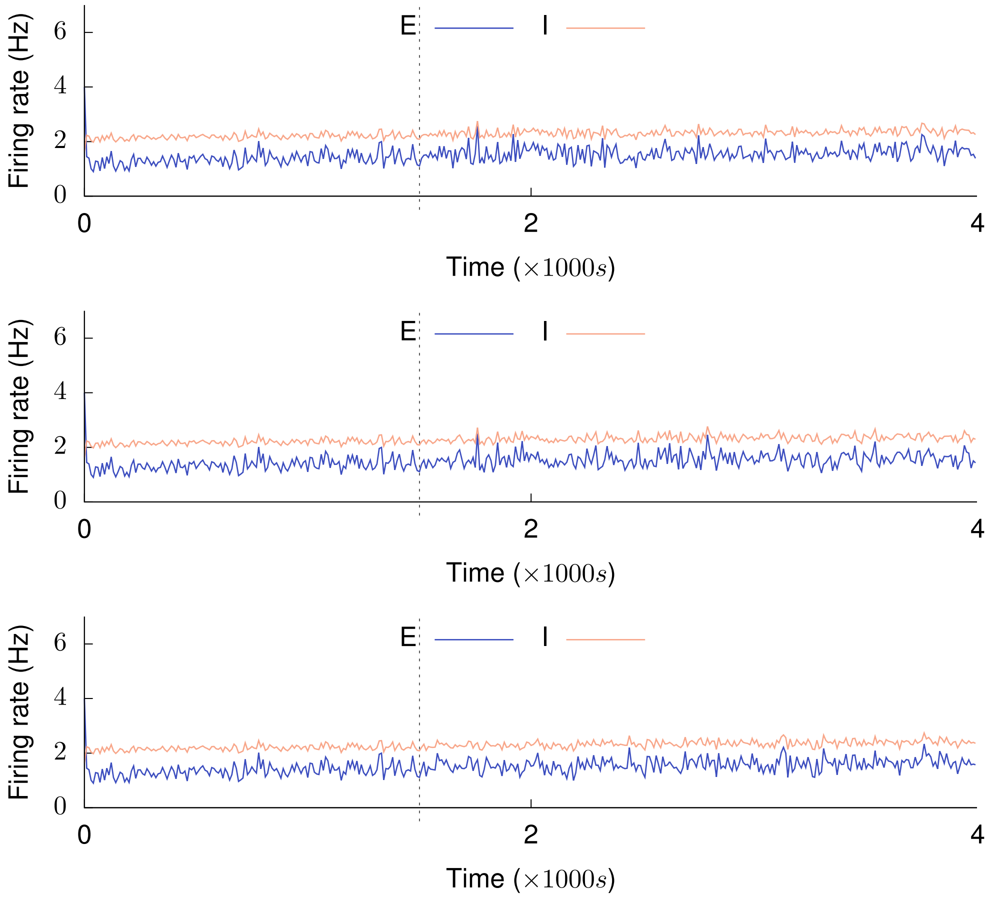

Figure 2: Mean firing rates for E and I neurons from three simulations with identical seeds but different structural plasticity update intervals. Synaptic plasticity is always active. Structural plasticity is enabled at $t = 2000$ s (dashed line). (The graphs are identical for $t < 2000$ s because the same seeds have been used in them.)

*References

Börgers, Christoph and Nancy Kopell (Mar. 2003). "Synchronization in Networks of Excitatory and Inhibitory Neurons with Sparse, Random Connectivity". In: *Neural Computation* 15.3, pp. 509–538. DOI: 10.1162/089976603321192059.

Brunel, Nicolas (2000). "Dynamics of sparsely connected networks of excitatory and inhibitory spiking neurons". In: *Journal of computational neuroscience* 8.3, pp. 183–208. ISSN: 1573-6873. DOI: 10.1023/A:1008925309027.

Butz, M., Ines D. Steenbuck, and A. van Ooyen (2014a). "Homeostatic structural plasticity can account for topology changes following deafferentation and focal stroke". In: *Frontiers in Neuroanatomy* 8, p. 115. DOI: 10.3389/fnana.2014.00115.

– (2014b). "Homeostatic structural plasticity increases the efficiency of small-world networks". In: *Frontiers in synaptic neuroscience* 6. DOI: 10.3389/fnsyn.2014.00007.

Butz, M. and A. van Ooyen (2013). "A Simple Rule for Dendritic Spine and Axonal Bouton Formation Can Account for Cortical Reorganization after Focal Retinal Lesions". In: *PLoS Comput Biol* 9.10, e1003259. DOI: 10.1371/journal.pcbi.1003259.

– (2014). "Homeostatic structural plasticity–a key to neuronal network formation and repair". In: *BMC Neuroscience* 15.Suppl 1, P17. DOI: 10.1186/1471-2202-15-s1-p17.

Butz, M., Arjen Van Ooyen, and Florentin Wörgötter (2009). "A model for cortical rewiring following deafferentation and focal stroke". In: *Frontiers in Computational Neuroscience* 3. DOI: 10.3389/neuro.10.010.2009.

Chen, Jerry L. et al. (2012). "Clustered dynamics of inhibitory synapses and dendritic spines in the adult neocortex". In: *Neuron* 74.2, pp. 361–373. DOI: 10.1016/j.neuron.2012.02.030.

Deger, Moritz et al. (2012). "Spike-timing dependence of structural plasticity explains cooperative synapse formation in the neocortex". In: *PLoS computational biology* 8.9, e1002689. DOI: 10.1371/journal.pcbi.1002689.

Destexhe, Alain and Denis Paré (Apr. 1999). "Impact of Network Activity on the Integrative Properties of Neocortical Pyramidal Neurons In Vivo". In: *Journal of Neurophysiology* 81.4, pp. 1531–1547. DOI: 10.1152/jn.1999.81.4.1531.

Destexhe, Alain, Michael Rudolph, and Denis Paré (2003). "The high-conductance state of neocortical neurons in vivo". In: *Nature Reviews Neuroscience* 4.9, pp. 739–751. DOI: 10.1038/nrn1198. URL: https://www.nature.com/articles/nrn1198.

Evarts, Edward V. (Mar. 1964). "TEMPORAL PATTERNS OF DISCHARGE OF PYRAMIDAL TRACT NEURONS DURING SLEEP AND WAKING IN THE MONKEY". In: *Journal of Neurophysiology* 27.2, pp. 152–171. DOI: 10.1152/jn.1964.27.2.152.

Golomb, D. and D. Hansel (May 2000). "The Number of Synaptic Inputs and the Synchrony of Large, Sparse Neuronal Networks". In: *Neural Computation* 12.5, pp. 1095–1139. DOI: 10.1162/089976600300015529.

Hubel, D. H. (Sept. 1959). "Single unit activity in striate cortex of unrestrained cats". In: *The Journal of Physiology* 147.2, pp. 226–238. DOI: 10.1113/jphysiol.1959.sp006238.

Keck, Tara, Thomas D. Mrsic-Flogel, et al. (2008). "Massive restructuring of neuronal circuits during functional reorganization of adult visual cortex". In: *Nature neuroscience* 11.10, pp. 1162–1167. DOI: 10.1038/nn.2181.

Keck, Tara, Volker Scheuss, et al. (2011). "Loss of sensory input causes rapid structural changes of inhibitory neurons in adult mouse visual cortex". In: *Neuron* 71.5, pp. 869–882. ISSN: 0896-6273. DOI: 10.1016/j.neuron.2011.06.034. URL: http://www.sciencedirect.com/science/article/pii/S0896627311005642.

Knott, Graham W. et al. (2002). "Formation of dendritic spines with GABAergic synapses induced by whisker stimulation in adult mice". In: *Neuron* 34.2, pp. 265–273. DOI: 10.1016/s0896-6273(02)00663-3.

Kunkel, Susanne et al. (2017). *NEST 2.12.0*. DOI: 10.5281/zenodo.259534.

Marik, Sally A. et al. (2010). "Axonal dynamics of excitatory and inhibitory neurons in somatosensory cortex". In: *PLoS Biology* 8.6, e1000395. DOI: 10.1371/journal.pbio.1000395.

Nowotny, T. and R. Huerta (Oct. 2003). "Explaining synchrony in feed-forward networks:" in: *Biological Cybernetics* 89.4, pp. 237–241. DOI: 10.1007/s00422-003-0431-9.

Papadopoulou, M. et al. (May 2011). "Normalization for Sparse Encoding of Odors by a Wide-Field Interneuron". In: *Science* 332.6030, pp. 721–725. DOI: 10.1126/science.1201835.

Qu, Jingyi et al. (Sept. 2013). "Oscillations and synchrony in a cortical neural network". In: *Cognitive Neurodynamics* 8.2, pp. 157–166. DOI: 10.1007/s11571-013-9268-7.

Schuemann, Anne et al. (2013). "Structural plasticity of GABAergic axons is regulated by network activity and GABAA receptor activation". In: *Frontiers in neural circuits* 7, p. 113. DOI: 10.3389/fncir.2013.00113.

Steriade, Mircea (1978). "Cortical long-axoned cells and putative interneurons during the sleep-waking cycle". In: *Behavioral and Brain Sciences* 1.3, pp. 465–485. DOI: 10.1017/S0140525X00076111. URL: https://doi.org/10.1017/S0140525X00076111.

Turrigiano, Gina G. (1999). "Homeostatic plasticity in neuronal networks: the more things change, the more they stay the same". In: *Trends in neurosciences* 22.5, pp. 221–227. DOI: 10.1016/s0166-2236(98)01341-1.

van Ooyen, A. and M. Butz (2017). *The rewiring brain*. Academic Press. ISBN: 9780128038727. URL: https://www.elsevier.com/books/the-rewiring-brain/van-ooyen/978-0-12-803784-3.

Vogels, T. P. et al. (2011). "Inhibitory plasticity balances excitation and inhibition in sensory pathways and memory networks". In: *Science* 334.6062, pp. 1569–1573. DOI: 10.1126/science.1211095. URL: http://www.sciencemag.org/content/334/6062/1569.short.

Wierenga, Corette J., Nadine Becker, and Tobias Bonhoeffer (2008). "GABAergic synapses are formed without the involvement of dendritic protrusions". In: *Nature neuroscience* 11.9, p. 1044. DOI: 10.1038/nn.2180.

Yamahachi, Homare et al. (2009). "Rapid axonal sprouting and pruning accompany functional reorganization in primary visual cortex". In: *Neuron* 64.5, pp. 719–729. DOI: 10.1016/j.neuron.2009.11.026.
