# Supplementary material for: Growth rules for the repair of Asynchronous Irregular neuronal networks after peripheral lesions
Source: PLoS Comput Biol. 2021 Jun 1;17(6):e1008996. doi: 10.1371/journal.pcbi.1008996 (PMC8195387; doi:10.1371/journal.pcbi.1008996)
Supplement: Attachment — Submitted filename: Re-Review.pdf [file pcbi.1008996.s003.pdf]

Reviewer #2

blue = first review | black = authors' reply | red = second review

The manuscript addresses possible mechanisms of repair in neuronal networks that are partially deprived of their input, for example as a consequence of peripheral lesions. The scientific scope of the work is very similar to a paper published in PLOS Computational Biology many years ago (Butz & van Ooyen, 2013). The core concept of the solution proposed in the new manuscript – homeostatic structural plasticity – was in fact brought up in several papers by Arjen van Ooyen and colleagues around the year 2009. The claim of the new manuscript is that the original idea does not work any more in the biologically more realistic setting of asynchronous-irregular (AI) network activity. Imposing additional functional plasticity to all inhibitory synapses in the network, however, is found to mitigate these problems, and successful repair could be demonstrated in numerical simulations.

In view of the strong overlap in concepts and results of the new submission and a previous publication, the question arises, whether the novelty represented by the submitted manuscript deserves a separate publication of it at all. For that reason, I will discuss strengths and weaknesses of the new manuscript in comparison to the previous publication.

Thank you very much for your review.
We would like to note that our manuscript does not suggest that the original idea of homeostatic structural plasticity does not work. Indeed, our post-synaptic growth curves are homeostatic as they maintain the activity of the neuron at its optimal level. Our results show that the particular configuration of growth curves proposed by Butz and van Ooyen (2013) do not permit network repair in the biologically plausible cortical network model by Vogels et al. (2011) that we use in our simulations. Thus, we follow the same method of reproducing characteristics of repair after deafferentation as Butz and van Ooyen (2013) to investigate and suggest new configurations of growth curves required for repair in this network model. This does not contradict previous work. We have made a number of changes to the manuscript to ensure that this is clearly stated in the text.
As pointed out by Reviewers 1 and 3, our work represents the next logical step in refining the growth curves by the inclusion of more biological realism. We believe that our results contribute to the understanding of structural plasticity and merit dissemination to the research community.

The “growth curves” described in the original simulations by Butz and van Ooyen constitute a phenomenological model of regulated structural plasticity. Tweaking these curves cannot increase the “biological realism” of the model. For this, one would have to address the question, for instance, how such a homeostatic controller is actually implemented in terms of sub-cellular signaling processes.

(1) In their introduction, the authors of the new study make the following statement: “Since the peripheral lesion model proposed by Butz and van Ooyen [33] was not based on a balanced cortical network model with biologically realistic AI activity, their hypothesised growth rules did not elicit repair in our simulations.” This claim provides justification to take up the issue again and propose a new solution to it. The logic of this statement, however, is problematic, as no analysis of sufficient generality was performed in the submitted manuscript. Whereas the authors of the original study used the Izhikevich neuron model, the new study employs an integrate-and-fire neuron model with conductance-based synapses. In both cases, 80% of all neurons were excitatory, and the remaining 20% inhibitory. The original study considered networks comprising 400 neurons in total, whereas the new study seems to have performed simulations of a 25-fold larger network, following Vogels et al. (2011). The actual numbers used are not revealed in the manuscript, however.

What then is the basis of the claim that the 2013 paper did not consider networks in the AI state? This issue was briefly addressed in Fig. 12 of the original publication, which displays only population activity traces, no spike trains. Although no formal analysis was performed, the visual appearance of the traces shown indeed suggests AI-like activity. Interestingly, the authors of the new paper do not provide any formal analysis of this issue, neither concerning their own networks, nor the networks of the old paper. The activity states of their simulated networks are not characterized quantitatively, and the spike trains shown in Fig. 4 of the submitted manuscript do not allow any conclusions either, they do not even have a proper time axis. Therefore, a convincing quantitative underpinning of the above-mentioned claim is inevitable.

Thank you for pointing out that we should have added a formal definition of the AI regime to our manuscript. We agree.

Since we use the cortical network model developed by Vogels et al. (2011) in our work, we also use the AI metric that was used by them in their work. They defined the AI state formally as:

$$(\text{ISI CV} > 1) \wedge (\sigma \text{ rate} < 5 \text{ Hz}) \quad (2)$$

where the ISI CV is the mean coefficient of variation of the inter-spike intervals (ISI) of neurons, and σ rate is the standard deviation of the population firing rate. We have added this definition of the AI state to the manuscript as Equation 17. The raster plots in Figure 4, as stated in the caption, cover a 1 second interval.

I find this definition quite problematic, as it addresses the degree of neuronal synchrony only very indirectly. Synchrony, however, has a big impact on correlation-based learning rules (like STDP), so it should be tracked very carefully in a model that is using STDP as one of its components.

Since neither the source code from Butz and van Ooyen (2013), nor the data generated from the work has been made publicly available, and additionally, since the firing characteristics of the network were not discussed in detail, it is difficult to provide objective evidence about the firing regime in the network. We can, therefore, only estimate some related metrics. In Figure 12 A, in the first panel that represents the normal network before deafferentation, there are approximately 10 neurons firing in every 1ms bin shown, for 1000 ms—a total of 10,000 spikes in the 1 second window. These figures include a total of 70 LPZ + 97 peri-LPZ = 167 neurons. Given that the network as a 4:1 ratio of E:I neurons, we approximate that this includes (4/5 of 167) = 130 E neurons. The mean population firing rate can thus be inferred to be: 10,000/130 ≈ 75 Hz. Spontaneous firing of cortical neurons, however, has been observed at much lower rates (< 20 Hz (Evarts 1964; Destexhe and Pare 1999; Hubel 1959; Steriade 1978)).

I really do not understand the issue here. Gallinaro and Rotter (Sci Rep, 2018) considered networks of LIF neurons with homeostatic structural plasticity. Imposing low firing rates for all neurons (set point at 8 Hz), they did not report any difficulty establishing robust AI activity. You might want to include this paper in your reference list anyway.

As noted above, since a complete analysis of the network firing regime in Butz and van Ooyen (2013) cannot be carried out, we have amended the introductory text to read as follows:

> Access to such data and recent advances in simulation technology have enabled computational modelling of activity dependent structural plasticity (Butz, VanOoyen, and Wörgötter 2009; Deger et al. 2012; Butz and van Ooyen 2013; Butz and van Ooyen 2014; Butz, Steenbuck, and van Ooyen 2014a; Butz, Steenbuck, and van Ooyen 2014b; van Ooyen and Butz 2017). In their seminal work, Butz and van Ooyen introduced the MSP framework (Butz, VanOoyen, and Wörgötter 2009). They demonstrated its utility by simulating a peripheral lesioning study to explore the activity dependent growth rules of neurites (Butz and van Ooyen 2013; Butz and van Ooyen 2014). Their analysis suggests that the restoration of activity could only be caused by the experimentally noted inward increase in excitatory lateral projections into the LPZ when dendritic elements sprouted at a lower level of activity than their axonal counterparts. Further, since excitatory and inhibitory synaptic elements were treated identically in their model, and this results in inhibitory projections also flowing into the LPZ instead of growing outwards from the LPZ, Butz and van Ooyen also discuss that the contribution of inhibitory neurons to the repair process remains an important open question. A computational model of peripheral lesioning that reproduces all features of the repair process in cortical networks is therefore still lacking.
>
> Here, as the next step towards improving our understanding of activity dependent structural plasticity in cortical networks, we build on Butz and van Ooyen's work to re-investigate activity dependent growth rules for neurites in the biologically plausible cortical network model developed by Vogels, Sprekeler et al. (Vogels et al. 2011). Unlike in (Butz and van Ooyen 2013) where the cortical network to be deafferented was "grown" using a pre-set free parameter, the Vogels, Sprekeler network model explicitly incorporates cortical network characteristics. Additionally, it is also balanced by homeostatic inhibitory STDP to a low frequency AI (spontaneous) firing regime as observed in the mammalian cortex (Brunel 2000; Destexhe, Rudolph, and Pare 2003) and has been demonstrated to function as an attractor-less store for associative memories (Vogels et al. 2011). By deafferenting this network and reproducing the course of repair as reported in experimental work, we systematically derive activity dependent growth rules for all neurites—excitatory and inhibitory, pre-synaptic and post-synaptic.

As already discussed in the manuscript, the growth rules predicted by our work do not conflict with the ones suggested by Butz and van Ooyen. Different networks with different characteristics may exhibit different growth rules:

Our simulation results do not imply that these are the only configurations of activity dependent growth rules that can underlie the turnover of neurites. Given the variety of neurons and networks in the brain, many configurations (and a variety of growth curves in each configuration) may apply to neurons. The results suggested here are hypothesized using an inhibition-balanced AI cortical network model, and so must be limited to such networks. As an example of a different configuration of growth curves that replicated repair in a different network model, Butz and van Ooyen's simulations proposed that all neurites are sprouted when neurons have less than optimal activity, and that the condition necessary for repair by an ingrowth of excitatory connections is that dendritic elements should be formed before axonal ones (Butz and van Ooyen 2013).

…

The numbers of neurons, defined as NE and NI , are listed in Table 5 as 8000 and 2000 respectively. We have now also added a reference to Table 5 in the caption of Figure 1.

(2) The authors of the new study claim that the hypothesized growth rules did not elicit repair in their simulations. This statement is also very problematic. Maybe the authors just have not tried hard enough to get it to work. Many parameters are different compared to the original setting, so it would not be surprising if also some of the parameters of the plasticity rule need adjustment. The only acceptable argument why it cannot work would be based on a mathematical analysis of the situation, but no such analysis is provided in the manuscript. Instead, the authors suggest an entirely new component of the model (inhibitory STDP) to make network repair possible. This scenario could be accepted as one possible ("sufficient") solution to the problem. In their abstract, however, the authors make the claim that their solution is the only one possible ("necessary"). They claim "Lastly, we observe that our proposed model of homeostatic structural plasticity and the inhibitory synaptic plasticity mechanism that also balances our AI network are both necessary for successful rewiring of the network." No result presented in the manuscript can support this claim. This issue needs clarification.

Thank you for raising this issue. The configurations of growth curves suggested by the model are indeed necessary for repair in our model. This arises from the differential effects of deafferentation on neurons in and outside the LPZ. As shown in the manuscript, whereas the activity of neurons in the LPZ does drop as a result of deafferentation as expected, the activities of neurons outside it increases—a net loss of inhibition. Thus, neurons outside the LPZ must lose excitatory inputs and gain inhibitory ones to reduce their activity. The growth rules proposed in Butz and van Ooyen (2013) do not allow this. Based on these growth rules, an increase in activity beyond the homeostatic level results in a retraction of all neurites—excitatory or inhibitory, pre-synaptic or post-synaptic. It was this observation that led us to investigate new growth rules. We have clarified the relevant text in the discussion to clearly state that the growth rules proposed here are necessary for repair in this particular inhibition-balanced cortical network setup, but that other growth rules like the ones proposed in Butz and van Ooyen (2013) may apply to other networks.

We agree that in the absence of a complete parameter search, which is not currently tractable due to the high computational costs of these simulations, the particular growth curves used in the paper represent only one possible set of parameters. We have updated the manuscript in multiple locations to stress that the particular configuration of growth rules is necessary, but not the particular set of parameters governing the growth curves used here. We note that this modelling methodology is very much in line with that used in Butz and van Ooyen (2013), where the exact parameters governing the growth curves (ηD , ηA , ε) are not derived by a parameter search or mathematical analysis. Instead, perhaps also limited by the computational costs of running a complete parameter search, the required configurations are also obtained there by matching the course of repair in simulations to experimental observations and elimination of other configurations that lead to "aberrant network reorganization". The general issue of a lack of tools for efficient modelling of structural plasticity that will make complete parameter searches tractable has been discussed in our manuscript.

Our simulations show that after deafferentation, in the presence of only structural plasticity, the network does undergo rewiring to return activity to deprived neurons in the LPZ. However, in our model, activity does not re-balance to a low firing AI state if synaptic plasticity is not present. As documented, this indicates that the larger, discrete changes made by structural plasticity to synaptic conductances are insufficient to balance excitation and inhibition in the network. The much smaller tweaks made by synaptic plasticity are necessary to finely tune inhibitory conductances to achieve this balance. The claim in the abstract is, there- fore, supported by our results in the scope of this modelling study.

Please make sure the logic of your argument is correct. As I understand it, by trial-and-error you found a solution that includes inhibitory STDP, fine. So this specific combination of homeostatic structural plasticity and inhibitory STDP is sufficient to get stable repair. This combination, however, cannot be claimed to be a necessary prerequisite. Without proper mathematical analysis, any such claim would be logically wrong.

(3) The new solution proposed in the manuscript involves two different types of synaptic plasticity, homeostatic structural plasticity and inhibitory spike-timing dependent plasticity. Whereas the latter is relatively fast with time scales in the range of tens of milliseconds, the former is rather slow, operating on time scales that are several orders of magnitude larger. But how then can fast inhibitory plasticity compensate for the deficits of slow homeostatic plasticity? On page 9, second to last paragraph the authors state: “Simulations require the growth rates of inhibitory axonal elements to be high enough to stabilise the large number of hyperactive neurons outside the LPZ.” It remains unclear why inhibitory plasticity cannot compensate for this.

Generally, the new manuscript does not provide sufficient insight into the question how the two types of plasticity interact. For example, a simple tracking of inhibitory amplitudes in the course of time, together with structural changes that happen simultaneously, would shed light on this question. Such extra insight is absolutely necessary to justify a solution that is considerably more complicated than previous suggestions.

Thank you for this comment. As discussed above, the model deals with two types of plasticity, one synaptic and one structural. Synaptic plasticity was originally included as it is a feature of the cortical network model by Vogels et al. (2011) - it can only modulate the strengths of already existing synapses, but it cannot create or remove synapses. The restoration of excitation to the deprived neurons in the LPZ requires the formation of new synapses, which the structural plasticity mechanism creates. Similarly for inhibition, the inhibitory STDP mechanism only modulates efficacies of synapses that already exist. The structural plasticity mechanism is necessary to create new inhibitory synapses to inhibit neurons. Once these new inhibitory synapses have been created, the inhibitory STDP mechanism is able to modulate them to balance both local and global excitation with inhibition. Thus, while the two types of plasticity modify the network at different temporal and spatial scales, they both contribute to the balancing of excitation and inhibition in the network.

I believe it when I see it. In the present version of your manuscript, no data are shown to make this interpretation plausible. Tracking inhibitory amplitudes in the course of time, as suggested previously, would be appropriate.

(4) The authors of the submitted manuscript discuss different options for the growth curves describing the homeostatic controller. However, they discuss only growth curves that arise from a translation of the Gaussian curve in x and y direction. What is the exact motivation of this somewhat restricted perspective? As the most important parameter is the slope of the curve in the set-point, other transformations (e.g. a stretching) of the growth curves actually seem more relevant. Why does one need a Gaussian shape for the axonal elements to begin with? Wouldn't the creation of axonal elements at a constant rate provide a simpler (better) solution? A more systematic account of these questions is necessary to go beyond the insight from previous studies.

Thank you for this comment. The use of Gaussian growth curves is a feature of the MSP framework. The work presented here makes use of the MSP framework to investigate structural plasticity. Investigations of other families of growth curves beyond the MPS is, unfortunately, beyond the scope of our work, given the computational costs associated with it.

The necessary condition that we apply to derive the growth curves is that at the set-point (the optimal activity of the neuron, ψ), no change in neurites should occur in neurons ($dz/dt = 0$ for all neurites). By reproducing the course of repair from experiments, we are able to ascertain which of the two fixed points of each Gaussian growth curve, where $dz/dt = 0$ holds, should take the value of ψ. As discussed above, a systematic variation of the parameters of the growth curves (which would stretch or squeeze them in x and y directions) is intractable with the current research technologies and so, we limit our results to the prediction of configurations of the activity dependent growth curves only. The exact parameters for growth curves, which may vary for different cell types, remains to be ascertained.

As stated previously, the most important parameter is the slope of the growth curve in the set-point. In particular, the set-point with a positive slope is unstable, and the corresponding firing rate will not be observed in equilibrium.

Furthermore, our preliminary simulations showed that constant availability of axonal contacts would not reproduce the characteristics of the course of repair observed in experiments. If excitatory axonal points are available to the deprived neurons in the LPZ from themselves, since synapse formation is distance dependent and more likely between neurons closer together, they will be prevented from forming synapses with free axonal neurites on neurons outside the LPZ that are further away—no longer reproducing the inward sprouting of excitatory axons to the LPZ. Similarly, if inhibitory axons are always being formed, neurons outside the LPZ that have more activity than necessary will form inhibitory synapses with other nearby neurons outside the LPZ instead of the more distant neurons in the LPZ—no longer reproducing the outgrowth of inhibitory axons from the LPZ.

In general, we attempted to remain as faithful to Butz and van Ooyen's MSP as possible to limit the scope of the work to the derivation of activity dependent dynamics that allow repair in the model following the course observed in experiments.

I think this would be relevant information for the readers of your paper. It reveals important aspects of the inner workings of homeostatic structural plasticity. I would recommend to perform not only preliminary, but systematic simulations with this variant of the rule and add it to the manuscript.
