# Supplementary material for: Growth rules for the repair of Asynchronous Irregular neuronal networks after peripheral lesions
Source: PLoS Comput Biol. 2021 Jun 1;17(6):e1008996. doi: 10.1371/journal.pcbi.1008996 (PMC8195387; doi:10.1371/journal.pcbi.1008996)
Supplement: Attachment — Submitted filename: 20210407-SinhaEtAl-review-responses-2.pdf [file pcbi.1008996.s004.pdf]

UH Biocomputation Group,
University of Hertfordshire,
Hatfield AL10 9AB,
UK.
April 7, 2021

The Editors,
PLOS CB.

Dear Madam/Sir,

Thank you very much for your consideration.

We have made a number of modifications to the manuscript to address the remaining issues. We note the limits of our numerical method, and point out that whereas the two plasticity mechanisms contribute to repair in our simulations, their precise individual roles require further theoretical analyses for clarification. We also include an additional measure of synchrony in the network, along with additional simulations, figure panels and information as requested by the reviewers.

We would like to thank the reviewers for their continued feedback.

Please find attached:

- a revised version of the manuscript,
- a version of the manuscript indicating the changes that we have made.

Reviewer 2

Colour code:

- Blue: first response,
- Red: second review,
- Black: second response.

Thank you very much for your review.

We would like to note that our manuscript does not suggest that the original idea of homeostatic structural plasticity does not work. Indeed, our post-synaptic growth curves are homeostatic as they maintain the activity of the neuron at its optimal level. Our results show that the particular configuration of growth curves proposed by Butz and van Ooyen (2013) do not permit network repair in the biologically plausible cortical network model by Vogels et al. (2011) that we use in our simulations. Thus, we follow the same method of reproducing characteristics of repair after deafferentation as Butz and van Ooyen (2013) to investigate and suggest new configurations of growth curves required for repair in this network model. This does not contradict previous work. We have made a number of changes to the manuscript to ensure that this is clearly stated in the text.

As pointed out by Reviewers 1 and 3, our work represents the next logical step in refining the growth curves by the inclusion of more biological realism. We believe that our results contribute to the understanding of structural plasticity and merit dissemination to the research community.

The “growth curves” described in the original simulations by Butz and van Ooyen constitute a phenomenological model of regulated structural plasticity. Tweaking these curves cannot increase the “biological realism” of the model. For this, one would have to address the question, for instance, how such a homeostatic controller is actually implemented in terms of sub-cellular signaling processes.

Thank you very much for your review.

We agree with your comment. The manuscript notes that we use the same investigative method that was used by Butz and van Ooyen to re-investigate the growth curves in a more biologically plausible cortical network model. We have updated the text to ensure that we do not claim that this results in more biologically-plausible growth curves.

Thank you for pointing out that we should have added a formal definition of the AI regime to our manuscript. We agree.

Since we use the cortical network model developed by Vogels et al. (2011) in our work, we also use the AI metric that was used by them in their work. They defined the AI state formally as:

$$(\text{ISI CV} > 1) \wedge (\sigma_{rate} < 5\,\text{Hz}) \tag{1}$$

where the ISI CV is the mean coefficient of variation of the inter-spike intervals (ISI) of neurons, and σ_{rate} is the standard deviation of the population firing rate. We have added this definition of the AI state to the manuscript as Equation 17. The raster plots in Figure 4, as stated in the caption, cover a 1 second interval.

I find this definition quite problematic, as it addresses the degree of neuronal synchrony only very indirectly. Synchrony, however, has a big impact on correlation-based learning rules (like STDP), so it should be tracked very carefully in a model that is using STDP as one of its components.

Thank you for your comment.

We have included an additional measure of synchrony—the average pairwise cross-correlation coefficient as used by Destexhe (2009)—in the manuscript as Figure 4B. We have also updated the methods section to detail how this metric is calculated.

Since neither the source code from Butz and van Ooyen (2013), nor the data generated from the work has been made publicly available, and additionally, since the firing characteristics of the network were not discussed in detail, it is difficult to provide objective evidence about the firing regime in the network. We can, therefore, only estimate some related metrics. In Figure 12 A, in the first panel that represents the normal network before deafferentation, there are approximately 10 neurons firing in every 1ms bin shown, for 1000 ms—a total of 10,000 spikes in the 1 second window. These figures include a total of 70 LPZ + 97 peri-LPZ = 167 neurons. Given that the network as a 4:1 ratio of E:I neurons, we approximate that this includes (4/5 of 167) = ˜130 E neurons. The mean population firing rate can thus be inferred to be: $10,000/130 \approx 75\,\text{Hz}$. Spontaneous firing of cortical neurons, however, has been observed at much lower rates ($< 20\,\text{Hz}$ (Evarts 1964; Destexhe and Paré 1999; Hubel 1959; Steriade 1978)).

I really do not understand the issue here. Gallinaro and Rotter (Sci Rep, 2018) considered networks of LIF neurons with homeostatic structural plasticity. Imposing low firing rates for all neurons (set point at 8 Hz), they did not report any difficulty establishing robust AI activity. You might want to include this paper in your reference list anyway.

Thank you for your comment. We have cited the paper in the manuscript.

It is difficult to comment on Gallinaro and Rotter (2018) without analysing their data in detail. There are considerable differences in the evolution of the connectivity of our two networks. Where our network is initialised with a 2% connection probability and both excitatory and inhibitory connectivities undergo relatively large changes, their network retains its inhibitory connectivity with a much higher connection probability of 10%. Thus, it is possible that their network does not see the same net increase in local recurrent excitation and coupling as is seen in our simulations, preventing it from gaining synchrony.

Thank you for raising this issue. The configurations of growth curves suggested by the model are indeed necessary for repair in our model. This arises from the differential effects of deafferentation on neurons in and outside the LPZ. As shown in the manuscript, whereas the activity of neurons in the LPZ does drop as a result of deafferentation as expected, the activities of neurons outside it *increases*—a net loss of inhibition. Thus, neurons outside the LPZ *must* lose excitatory inputs and gain inhibitory ones to reduce their activity. The growth rules proposed in Butz and van Ooyen (2013) do not allow this. Based on these growth rules, an increase in activity beyond the homeostatic level results in a retraction of all neurites—excitatory or inhibitory, pre-synaptic or post-synaptic. It was this observation that led us to investigate new growth rules. We have clarified the relevant text in the discussion to clearly state that the growth rules proposed here are necessary for repair in this particular inhibition-balanced cortical network setup, but that other growth rules like the ones proposed in Butz and van Ooyen (2013) may apply to other networks.

We agree that in the absence of a complete parameter search, which is not currently tractable due to the high computational costs of these simulations, the particular growth curves used in the paper represent only one possible set of parameters. We have updated the manuscript in multiple locations to stress that the particular configuration of growth rules is necessary, but not the particular set of parameters governing the growth curves used here. We note that this modelling methodology is very much in line with that used in Butz and van Ooyen (2013), where the exact parameters governing the growth curves (η_D, η_A, ϵ) are not derived by a parameter search or mathematical analysis. Instead, perhaps also limited by the computational costs of running a complete parameter search, the required configurations are also obtained there by matching the course of repair in simulations to experimental observations and elimination of other configurations that lead to "aberrant network reorganization". The general issue of a lack of tools for efficient modelling of structural plasticity that will make complete parameter searches tractable has been discussed in our manuscript.

Our simulations show that after deafferentation, in the presence of only structural plasticity, the network does undergo rewiring to return activity to deprived neurons in the LPZ. However, in our model, activity does not re-balance to a low firing AI state if synaptic plasticity is not present. As documented, this indicates that the larger, discrete changes made by structural plasticity to synaptic conductances are insufficient to balance excitation and inhibition in the network. The much smaller tweaks made by synaptic plasticity are necessary to finely tune inhibitory conductances to achieve this balance. The claim in the abstract is, therefore, supported by our results in the scope of this modelling study.

Please make sure the logic of your argument is correct. As I understand it, by trial-and-error you found a solution that includes inhibitory STDP, fine. So this specific combination of homeostatic structural plasticity and inhibitory STDP is sufficient to get stable repair. This combination, however, cannot be claimed to be a necessary prerequisite. Without proper mathematical analysis, any such claim would be logically wrong.

Thank you.

We agree that without theoretical analyses, the necessity of the two mechanisms cannot be commented upon. We have modified the manuscript to say so. We limit our statements to note that in our simulations they both contribute to repair and network stability, and that further theoretical work is needed to clarify their interactions.

Thank you for this comment. As discussed above, the model deals with two types of plasticity, one synaptic and one structural. Synaptic plasticity was originally included as it is a feature of the cortical network model by Vogels et al. (2011) - it can only modulate the strengths of already existing synapses, but it cannot create or remove synapses. The restoration of excitation to the deprived neurons in the LPZ requires the formation of new synapses, which the structural plasticity mechanism creates. Similarly for inhibition, the inhibitory STDP mechanism only modulates efficacies of synapses that already exist. The structural plasticity mechanism is necessary to create new inhibitory synapses to inhibit neurons. Once these new inhibitory synapses have been created, the inhibitory STDP mechanism is able to modulate them to balance both local and global excitation with inhibition. Thus, while the two types of plasticity modify the network at different temporal and spatial scales, they both contribute to the balancing of excitation and inhibition in the network.

I believe it when I see it. In the present version of your manuscript, no data are shown to make this interpretation plausible. Tracking inhibitory amplitudes in the course of time, as suggested previously, would be appropriate.

As noted above, we have modified the manuscript to note that the interactions between the two homeostatic mechanisms needs further study.

We have also added figures that show the mean synaptic weights of projections received by neurons in various parts of the network over the repair process (Figures 6C, 7C, 8C, and 9C). Figures 7 and 9 show the simultaneous action of the two mechanisms. In these Figures, the structural plasticity mechanism can be seen to increase the number of inhibitory projections received by the neurons and the synaptic plasticity can be observed to modulate their IE weights in parallel.

Thank you for this comment. The use of Gaussian growth curves is a feature of the MSP framework. The work presented here makes use of the MSP framework to investigate structural plasticity. Investigations of other families of growth curves beyond the MPS is, unfortunately, beyond the scope of our work, given the computational costs associated with it.

The necessary condition that we apply to derive the growth curves is that at the set-point (the optimal activity of the neuron, ψ), no change in neurites should occur in neurons ($dz/dt = 0$ for all neurites). By reproducing the course of repair from experiments, we are able to ascertain which of the two fixed points of each Gaussian growth curve, where $dz/dt = 0$ holds, should take the value of ψ. As discussed above, a systematic variation of the parameters of the growth curves (which would stretch or squeeze them in x and y directions) is intractable with the current research technologies and so, we limit our results to the prediction of configurations of the activity dependent growth curves only. The exact parameters for growth curves, which may vary for different cell types, remains to be ascertained.

As stated previously, the most important parameter is the slope of the growth curve in the set-point. In particular, the set-point with a positive slope is unstable, and the corresponding firing rate will not be observed in equilibrium.

Thank you.

We have updated the manuscript to note the importance of the slopes of the growth curves at the fixed points in the section on the derivation of post-synaptic growth curves, and the caption of Figure 5.

Furthermore, our preliminary simulations showed that constant availability of axonal contacts would not reproduce the characteristics of the course of repair observed in experiments. If excitatory axonal points are available to the deprived neurons in the LPZ from themselves, since synapse formation is distance dependent and more likely between neurons closer together, they will be prevented from forming synapses with free axonal neurites on neurons outside the LPZ that are further away—no longer reproducing the inward sprouting of excitatory axons to the LPZ. Similarly, if inhibitory axons are always being formed, neurons outside the LPZ that have more activity than necessary will form inhibitory synapses with other nearby neurons outside the LPZ instead of the more distant neurons in the LPZ—no longer reproducing the outgrowth of inhibitory axons from the LPZ.

In general, we attempted to remain as faithful to Butz and van Ooyen's MSP as possible to limit the scope of the work to the derivation of activity dependent dynamics that allow repair in the model following the course observed in experiments.

I think this would be relevant information for the readers of your paper. It reveals important aspects of the inner workings of homeostatic structural plasticity. I would recommend to perform not only preliminary, but systematic simulations with this variant of the rule and add it to the manuscript.

Thank you.

We have run additional simulations in this configuration and added the results in summary Figure 11 and Table 2.

Yours sincerely,

Ankur Sinha and Volker Steuber

*References

Butz, M. and A. van Ooyen (2013). “A Simple Rule for Dendritic Spine and Axonal Bouton Formation Can Account for Cortical Reorganization after Focal Retinal Lesions”. In: *PLoS Comput Biol* 9.10, e1003259. DOI: 10.1371/journal.pcbi.1003259.

Destexhe, Alain (2009). “Self-sustained asynchronous irregular states and Up–Down states in thalamic, cortical and thalamocortical networks of nonlinear integrate-and-fire neurons”. In: *Journal of computational neuroscience* 27.3, pp. 493–506. DOI: 10.1007/s10827-009-0164-4. URL: http://www.opensourcebrain.org/projects/31.

Destexhe, Alain and Denis Paré (Apr. 1999). “Impact of Network Activity on the Integrative Properties of Neocortical Pyramidal Neurons In Vivo”. In: *Journal of Neurophysiology* 81.4, pp. 1531–1547. DOI: 10.1152/jn.1999.81.4.1531.

Evarts, Edward V. (Mar. 1964). “TEMPORAL PATTERNS OF DISCHARGE OF PYRAMIDAL TRACT NEURONS DURING SLEEP AND WAKING IN THE MONKEY”. In: *Journal of Neurophysiology* 27.2, pp. 152–171. DOI: 10.1152/jn.1964.27.2.152.

Gallinaro, Júlia V. and Stefan Rotter (2018). “Associative properties of structural plasticity based on firing rate homeostasis in recurrent neuronal networks”. In: *Scientific reports* 8.1, p. 3754. DOI: 10.1038/s41598-018-22077-3.

Hubel, D. H. (Sept. 1959). “Single unit activity in striate cortex of unrestrained cats”. In: *The Journal of Physiology* 147.2, pp. 226–238. DOI: 10.1113/jphysiol.1959.sp006238.

Steriade, Mircea (1978). “Cortical long-axoned cells and putative interneurons during the sleep-waking cycle”. In: *Behavioral and Brain Sciences* 1.3, pp. 465–485. DOI: 10.1017/S0140525X00076111. URL: https://doi.org/10.1017/S0140525X00076111.

Vogels, T. P. et al. (2011). “Inhibitory plasticity balances excitation and inhibition in sensory pathways and memory networks”. In: *Science* 334.6062, pp. 1569–1573. DOI: 10.1126/science.1211095. URL: http://www.sciencemag.org/content/334/6062/1569.short.
